# Probing the antigenicity of hepatitis C virus envelope glycoprotein complex by high-throughput mutagenesis

**DOI:** 10.1371/journal.ppat.1006735

**Published:** 2017-12-18

**Authors:** Radhika Gopal, Kelli Jackson, Netanel Tzarum, Leopold Kong, Andrew Ettenger, Johnathan Guest, Jennifer M. Pfaff, Trevor Barnes, Andrew Honda, Erick Giang, Edgar Davidson, Ian A. Wilson, Benjamin J. Doranz, Mansun Law

**Affiliations:** 1 Department of Immunology and Microbiology, The Scripps Research Institute, La Jolla, CA, United States of America; 2 Department of Integrative Structural and Computational Biology, The Scripps Research Institute, La Jolla, CA, United States of America; 3 Integral Molecular, Inc., Philadelphia, PA, United States of America; 4 The Skaggs Institute for Chemical Biology, The Scripps Research Institute, La Jolla, CA, United States of America; Institut Pasteur, FRANCE

## Abstract

The hepatitis C virus (HCV) envelope glycoproteins E1 and E2 form a non-covalently linked heterodimer on the viral surface that mediates viral entry. E1, E2 and the heterodimer complex E1E2 are candidate vaccine antigens, but are technically challenging to study because of difficulties in producing natively folded proteins by standard protein expression and purification methods. To better comprehend the antigenicity of these proteins, a library of alanine scanning mutants comprising the entirety of E1E2 (555 residues) was created for evaluating the role of each residue in the glycoproteins. The mutant library was probed, by a high-throughput flow cytometry-based assay, for binding with the co-receptor CD81, and a panel of 13 human and mouse monoclonal antibodies (mAbs) that target continuous and discontinuous epitopes of E1, E2, and the E1E2 complex. Together with the recently determined crystal structure of E2 core domain (E2c), we found that several residues in the E2 back layer region indirectly impact binding of CD81 and mAbs that target the conserved neutralizing face of E2. These findings highlight an unexpected role for the E2 back layer in interacting with the E2 front layer for its biological function. We also identified regions of E1 and E2 that likely located at or near the interface of the E1E2 complex, and determined that the E2 back layer also plays an important role in E1E2 complex formation. The conformation-dependent reactivity of CD81 and the antibody panel to the E1E2 mutant library provides a global view of the influence of each amino acid (aa) on E1E2 expression and folding. This information is valuable for guiding protein engineering efforts to enhance the antigenic properties and stability of E1E2 for vaccine antigen development and structural studies.

## Author summary

The function and structure of the hepatitis C virus envelope glycoprotein complex E1E2 is poorly understood because of difficulties in producing pure and correctly folded proteins for biochemical and structural analysis. Here, we use monoclonal antibodies to non-overlapping epitopes on E1E2, as well as the CD81 co-receptor, to probe a complete alanine-scanning library of the E1E2 protein. This comprehensive binding study delineates the antigenic regions of E1E2. This information is valuable for understanding the folding of E1E2 and for vaccine antigen design efforts.

## Introduction

**Fig 1:**
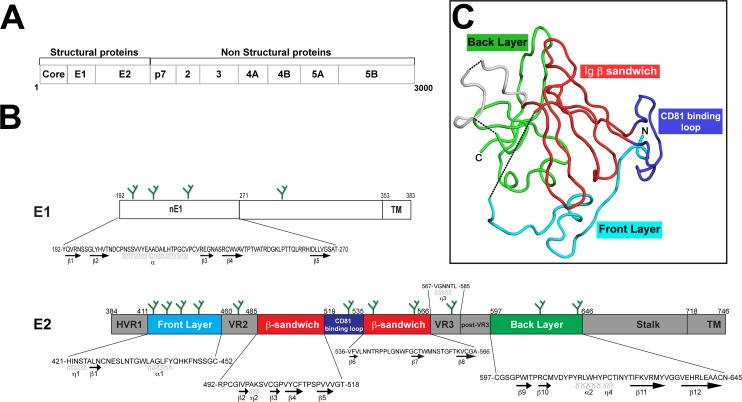
Overview of E1 and E2 glycoprotein structures. **(A)** The approximately 3000 amino-acid HCV polyprotein generates 10 proteins following cleavage, of which E1 and E2 are two of the three structural proteins. **(B)** Spanning amino acids 192–383, the structure of E1 is poorly understood although crystallization of the first half of the protein (aa192-271) revealed secondary structures that could be present in native E1 [19] including an α-helix flanked by several β-sheets. In contrast, E2 (aa 384–746) has several well-defined regions containing β-sheets, α-helices, and η (3_10_) helices. Sequences for regions in which secondary structure is known (e.g. nE1, E2 front layer, β-sandwich core, back layer, etc.) are included (prototypic H77 sequence). Green-branched forks depict relative locations of N-linked glycans. **(C)** Crystal structure of E2c [15] illustrated that E2 is characterized by a globular structure with a central Ig-like core flanked by front and back layers. The front layer, β-sandwich core, CD81 binding loop, and back layer are colored as depicted in panel **(B)**.

Hepatitis C virus (HCV) is a major global health concern with over 170 million people currently infected and an additional 3 million being infected each year (reviewed in [1, 2]). While approximately 30% of infected individuals are capable of spontaneously clearing the virus, usually within the first 12 months of infection, the remainder generally develops life-long infection. Of those who progress to chronic infection, about 20% develop liver cirrhosis and 1–3% hepatocellular carcinoma, one of the leading causes of cancer mortality [2, 3]. As a member of the *Hepacivirus* genus in the *Flaviviridae* family, HCV contains a positive-sense, single-stranded RNA genome coding for three structural proteins and seven non-structural proteins [4] (Fig 1A). The RNA-dependent RNA polymerase, NS5B, which lacks proofreading activity, gives rise to the heterogeneous viral quasispecies and the diverse viral genotypes in circulation. The high rate of infection in endemic countries and the morbidity caused by subsequent liver damage[5], as well as underdiagnosis, costly treatments and high rate of reinfection [6, 7], highlight the need for an effective HCV vaccine to limit virus infection and spread.

E1 and E2 are heavily glycosylated envelope proteins and form a heterodimer complex on the viral surface that facilitates viral attachment and entry into host cells (reviewed in [4]). E1 encompasses residues 192–383 of the HCV polyprotein (prototypic strain H77), while E2 is the larger of the two envelope proteins and spans amino acids 384–746 (Fig 1B). In association with apolipoproteins, HCV forms lipoviroparticles that attach and infect hepatocytes using a number of host entry factors including CD81, scavenger receptor class B member 1 (SR-B1), claudin-1, occludin, low-density lipoprotein receptor (LDLR), and others whose roles are still under investigation (reviewed in [4, 8]). CD81 was the first entry receptor identified and is the best-characterized entry factor to date. Many studies have demonstrated that CD81 is capable of binding to soluble E2, and antibodies targeting the large extracellular loop of CD81 (CD81-LEL) prevent HCV infection both *in vitro* and *in vivo* (reviewed in [9]). While E2 is known to interact with CD81 and SR-B1 (reviewed in [10]), it appears that E1 may help modulate these interactions and could play a role in membrane fusion [11–13].

Our understanding of the E2 protein has been enhanced by the recent crystallization of the E2 core domain (E2c), providing evidence that refutes the previous hypothesis of E2 as a class II fusion protein [14–16]. Canonical class II fusion proteins consist of three protein domains with an elongated structure in which domain 2 harbors the fusion peptide that is embedded within the dimer interfaces (reviewed in [17]). In contrast, E2 is globular in shape with the E2c adopting a compact architecture surrounded by disordered variable loops. E2c consists of a central Ig-like β-sandwich scaffold flanked by front and back layers of protein consisting of loops, short helices, and β-sheets [15, 16]. Cross-neutralizing antibodies that recognize E2c primarily map to the front layer, which is also a component of the CD81 binding site (CD81bs) [15]. Recent findings suggest that the CD81bs is exceptionally flexible in the soluble protein form and could present many non-optimal conformations during immunization [18]. Structural information for E1 is more limited, consisting of NMR studies of putative membrane proximal regions and a recent crystal structure of the N-terminal region, which displays an unusual disulfide-linked multimeric (nE1) organization [19].

Since E1 and E2 are the targets of neutralizing antibody (NAb) responses, understanding how they interact with antibodies can offer valuable insights into the antigenic surface and folding of the vaccine immunogens. To date, a number of antibodies targeting E1, E2, or the E1E2 complex have been isolated, some exhibiting cross-neutralizing behavior when tested against multiple viral genotypes (reviewed in [20–22]). The recent X-ray structures of E2c, E1 and E2 peptides complexed with several broadly neutralizing antibodies (bNAbs) have provided partial yet critical information on the architecture and functions of the glycoproteins [15, 16, 23–25]. To gain a greater understanding of the HCV envelope glycoprotein antigens, we exploited an alanine scanning mutant library spanning the entirety of E1 and E2, which had been recently created using a high-throughput shotgun mutagenesis method [26]. The comprehensive alanine scanning mutagenesis, in combination with antibodies recognizing a wide range of discontinuous epitopes, can provide a snapshot of how the different regions in E1 and E2 may be brought together to form the epitopes. We tested binding by a panel of 13 monoclonal antibodies (mAbs) and CD81-LEL fused to the immunoglobulin Fc fragment to probe the diverse epitopes encompassing distinct continuous and discontinuous antigenic sites on E1 and E2, thereby providing a global perspective of E1 and E2 antigenicity (Table 1, and Materials and Methods). Using high-throughput flow cytometry (FC), the effect of each point mutation on the binding of the antibodies and CD81-LEL was determined. The results were compared with data in the literature to uncover new information about the HCV epitopes. Selected mutations were analyzed further in complementary experiments to evaluate the mutagenesis results.

**Table 1:** Properties of the monoclonal antibody panel.

Glycoprotein	mAb	Antigenic site	Binding Region	Features
E1	A4 [28]	Continuous	197–207	Murine mAb; non-neutralizing
	IGH526 [23, 29]	Discontinuous	313–327	Weakly neutralizing, cross-reactive (1a, 1b, 4a, 5a, 6a)
E2	HCV1 [24, 30]	Continuous	412–423	L413, N415, G418, and W420 required for binding; potent, cross-reactive
	AP33 [25, 31, 32]	Continuous	412–423	Murine mAb; L413, N415, G418, and W420 required for binding; potent, cross-reactive
	AR1A/B [33]	Discontinuous	Non-neutralizing face of E2c	Strain specific; no neutralizing activity; AR1A can block CD81 binding to soluble E2
	AR2A [15, 18, 33]	Discontinuous	Back layer of E2c	Isolate-specific neutralization (1a, 2b, 4, 5)
	AR3A/B/C/D [15, 33]	Discontinuous	Neutralizing face of E2c	Potent, broadly neutralizing; overlaps the CD81 binding site
E1E2	AR4A [34]	Discontinuous	Not well defined	Neutralizes 6 genotypes in HCVpp and HCVcc systems
	AR5A [35]	Discontinuous	Not well defined	Neutralizes several strains (1a, 1b, 4, 5)

Our study revealed that several E2 back layer residues play a critical role in E1E2 folding and indirectly affect binding to CD81 and antigenic region (AR) 3 mAbs. The data also predict residues that are likely located at or near the interface for E1 and E2 complex formation.

## Results

### Residues essential for expression and folding of recombinant E1E2

**Fig 2:**
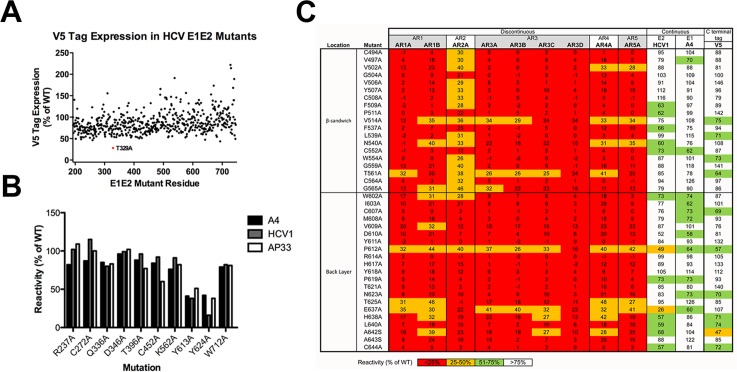
Identification of mutations that impair global folding. **(A)** The V5 tag present at the C-terminus of 545 mutants was used as a marker of overall E1E2 expression. The expression of the V5 tag for each mutant was normalized to V5 expression on wild-type E1E2 (left). Only one mutation T329A (red dot) resulted in markedly decreased V5 expression. **(B)** The expression of the ten remaining mutants not present in the library was assessed using antibodies targeting continuous epitopes as controls. A4 targets E1, and HCV1 and AP33 are specific for distinct but overlapping epitopes from E2 aa 412–423. **(C)** Numerous mutations resulted in less than 50% binding (relative to wild-type) to the panel of conformation-dependent antibodies (AR1-5) when analyzed by flow cytometry. These mutants were predominantly located in the central Ig-like β sandwich or the back layer of E2. Binding of HCV1 and A4, which recognize linear epitopes of E2 and E1, respectively, were included along with V5 tag expression. Coloring corresponds to the reactivity to each E1E2 mutant with <25% red, 25–50% orange, 51–75% green, and >75% white.

High-throughput flow cytometry is a well-established system that has been used in the study of many viral antigens, and therefore was applied to probe the E1E2 mutant library with a panel of mAbs and CD81-LEL [26]. The results combined with data generated by other methods (ELISA, immunoblots) in the literature are summarized in S1 Table. The E1E2 mutant library used here is comprised of 545 individual alanine mutations spanning nearly the entire E1E2 protein sequence of a genotype 1a isolate (H77, GenBank accession NC_004102). The remaining ten E1E2 mutations R237A, C272A, Q336A, D346A, T396A, C452A, K562A, Y613A, Y624A, and W712A were introduced into an equivalent E1E2 expression vector (plasmid H77c, [27]). The expression of the mutants was monitored by comparing mAb binding to wild-type E1E2 via the C-terminal V5 epitope fusion tag or mAbs to E1 and E2 continuous epitopes (A4, HCV1 and AP33). Only mutant T329A resulted in markedly reduced V5 expression compared to the other mutants in the library (Fig 2A); however, since a number of mAbs showed high levels of binding to the T329A mutant clone (S1 Table), it is possible that the V5 tag is partially occluded in this clone. The expression of the remaining 10 mutant constructs lacking the V5 tag was assessed by flow cytometry or ELISA based on their reactivity to the control mAbs (Fig 2B). Two mutations, Y613A and Y624A, resulted in reduced binding to control mAbs.

To identify mutations that influence global protein folding, we sought residues that, when mutated, resulted in less than 50% binding to mAbs targeting conformational epitopes on AR1-5 described in Table 1.

About 7% of residues (40 of 555) are important for global folding using these criteria (Fig 2C). All of these residues are present in E2, between amino acids (aa) 490–650, which form the central Ig scaffold and the back layer of E2c [15]. Mutations that resulted in greatly reduced E1E2 expression or improper global folding could not accurately be used for determining antibody epitopes and were excluded from subsequent analysis.

### Validation of high-throughput analysis using HCV1 and AP33

To help validate findings from flow cytometry-based evaluation of the E1E2 mutant library, we compared the flow cytometry results to previously published data that utilized site-directed mutagenesis and ELISA for the bNAbs HCV1 and AP33, both of which recognize the E2 antigenic site 412–423 (AS412) and have been extensively characterized [24, 25, 30–32, 36]. Residues critical for mAb binding were defined as those that retained >75% reactivity to one or more control mAbs, but also resulted in <25% binding to the mAb of interest upon mutation.

**Fig 3:**
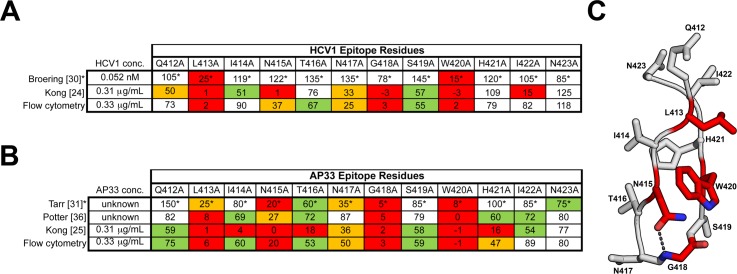
Flow cytometry confirms that residues 413–420 within the HCV1 and AP33 epitope are critical for binding to E2. The reactivity (as % of wild-type) of HCV1 **(A)** and AP33 **(B)** to E1E2 mutants suggested to encompass the HCV1 and AP33 epitope is shown. Previously published results are included, all of which utilized ELISA to measure reactivity. Coloring corresponds to the reactivity to each E1E2 mutant with <25% red, 25–50% orange, 51–75% green, and >75% white. For HCV1, our flow cytometry findings for L413 and W420 agree with previous reports. **(C)** The side chains of the amino acids in the E2 412–423 peptide are shown with the critical residues highlighted in red. *Values are approximated based on results originally published as a bar graph [30, 31].

The original mapping of mAb HCV1 resulted in the identification of a stretch of amino acids 412–423 following hypervariable region 1 (HVR1) of E2 as the epitope with L413 and W420 being critical for HCV1 binding [30]. The mapping approach used in this study correctly identified the critical residues L413, N415, G418, and W420 for both HCV1 and AP33, in overall agreement with previous mapping studies (Fig 3A and 3B [24, 25, 30, 31, 36]). Subsequent structural analysis revealed that N415 sidechain and G418 backbone amine form a hydrogen bond, stabilizing the β hairpin turn, required for both HCV1 and AP33 recognition [24, 25] (Fig 3C). These results show that the flow cytometry approach successfully identified critical residues for the well-characterized mAbs that target HCV linear epitopes.

### Epitope mapping of antibodies to E2 antigenic regions (ARs) 1 to 3

Previous studies used a small panel of alanine mutants and ELISA to define epitopes for seven mAbs, AR1A-B, AR2A, and AR3A-D, targeting three distinct antigenic regions on E2 [33]. Recently, co-crystallization of E2c with the Fab portion of the AR3C antibody offered a structural explanation for some of the mapping data [15].

**Fig 4:**
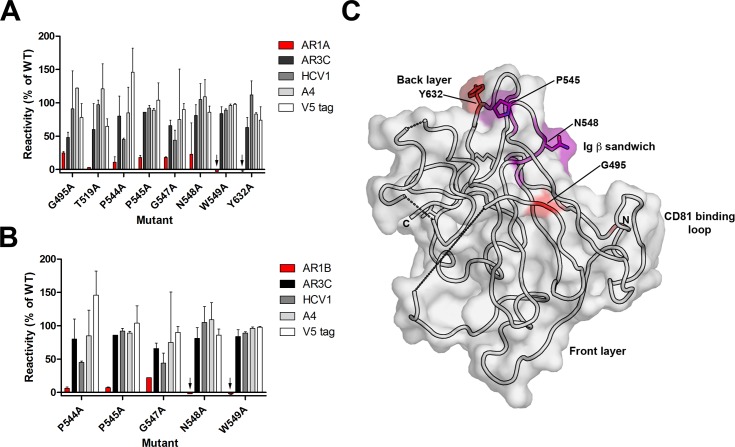
Epitopes for AR1 monoclonal antibodies target the non-neutralizing face of E2c. Single residue mutations which resulted in ≤25% of mAb binding (relative to wild-type E2) but >75% for at least one control antibody are shown for mAbs AR1A **(A)** and AR1B **(B)**. Binding assays were performed twice with the range indicated. Black arrows indicate negative values. **(C)** The critical residues for AR1A and AR1B were visualized on the E2c structure [15]. Residues in purple are required by both AR1A and AR1B. Residues specific for AR1A alone are in red. Dashed lines represent regions of E2c that are disordered or missing.

AR1A and AR1B have previously been described as binding genotype-specific E2 but lack significant neutralizing capabilities, suggesting that their epitopes are exposed on isolated E1E2 but not on the viral surface [33]. As expected, in our screening, mAbs AR1A and AR1B required many of the same residues for binding, confirming that these two antibodies recognize distinct but overlapping epitopes. Eight residues were found to be important for binding of mAb AR1A to E2 (Fig 4A) while five of these residues were also required for AR1B binding (Fig 4B). When visualized on the E2c structure (Fig 4C), residues recognized by both AR1A and AR1B are localized to a pocket near the top of E2c that is formed from the outer layer of the β sandwich in the previously described non-neutralizing face of E2c [15]. The remaining residues that are important for AR1A binding—G495, T519, and Y632—are located on the periphery of the pocket and may play an indirect role in binding. While both mAbs target the same antigenic region, only AR1A is capable of blocking CD81 binding to E2 [33]. Indeed, our results confirm that mutations of the epitope residues shared between mAbs AR1A and AR1B did not alter binding of CD81-LEL, while mutations of the three residues specific for AR1A resulted in decreased CD81 binding (S1 Table). The complete library scanning analysis also helped reassign the roles of several critical residues identified in previous mapping experiments (S2A Table). For example, two residues, V538 and N540, were originally thought to play a role in AR1A and AR1B binding [33]. However, flow cytometry results indicate that mutation of these two residues to alanine disturbed the global folding of E1E2, thereby resulting in loss of binding of these mAbs.

**Fig 5:**
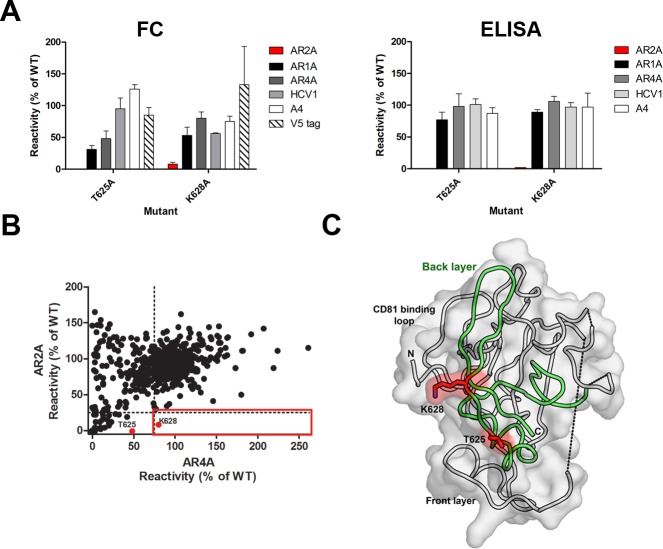
mAb AR2A binds the back layer of E2. **(A)** Data shown are the mean reactivities determined by flow cytometry (FC) and ELISA of mAb AR2A, conformational mAbs AR1A and AR4A, and linear mAbs HCV1, AP33, and A4 to mutants T625A and K628A. The C-terminal V5 tag expression is also shown for the flow cytometry constructs. Each binding assay was performed twice with the range shown. **(B)** The mean binding value (percent relative to wild-type reactivity) of mAb AR2A to each mutant E1E2 library clone was plotted as a function of binding to conformational-dependent mAb AR4A (black circles). Clones with AR2A binding ≤25%, but at least 75% AR4A binding (red boxes), are considered to indicate crucial residues for AR2A. As shown, only K628A (red circle) fell within this threshold. **(C)** Back-layer residues T625 and K628 are highlighted in red on the E2c structure [15]. The back layer is shown in green.

For AR2A, ELISA screening against a panel of back layer mutants showed that T625A and K628A reduced AR2A binding to less than 15% of wild-type reactivity, but with >75% binding for mAbs AR1A, AR1B, AR4A and AR5A (Fig 5A, S2B Table and [18]). Flow cytometry analysis showed that only K628A exhibited less than 25% AR2A binding and greater than 75% binding for AR4A control mAb (Fig 5B). We note that the T625A mutant was not identified as important by flow cytometry likely due to differences in antigen presentation between the assays (see discussion section). AR3 mAbs were excluded as controls because of the effect of back layer mutations on AR3 mAb binding (see below). Negative-stain EM reconstructions from Kong *et al*. [15] and the ELISA and flow cytometry mapping data that we report here strongly suggests that the back layer of E2 is involved in AR2A recognition (Fig 5C).

**Fig 6:**
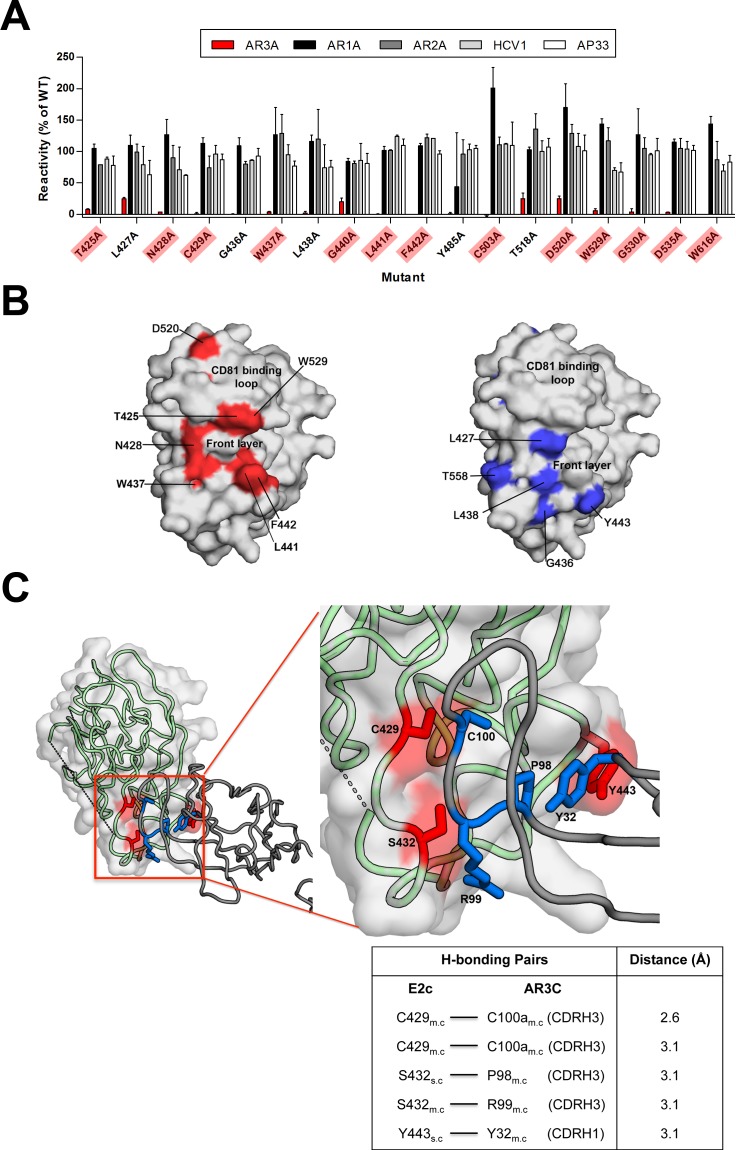
AR3 mAbs target overlapping but distinct epitopes on the neutralizing face of E2c. **(A)** Data shown are the mutated residues for which binding for mAb AR3A was ≤25%, but >75% for at least one control mAb. Any mutations that resulted in global misfolding (see **Fig 2B**) were excluded. Mutations leading to ≤25% binding for all four AR3 mAbs are highlighted in red. **S2 Fig** summarizes data for AR3B-3D mAbs. The percent reactivity is the mean of two experiments. **(B)** Seven out of thirteen critical residues for the four AR3 mAbs (highlighted in red in **A**) are shown in red on the E2c structure [15]. The remaining 6 residues are hidden in this orientation. Critical residues that are variable between AR3 mAbs are indicated in blue in the right panel. The exact location of C459 could not be visualized as aa 453–459 are disordered in the E2c structure. **(C)** The E2c-AR3C Fab crystal structure and hydrogen bonding analysis confirmed that several amino acids in the region identified by flow cytometry are likely involved in hydrogen bonding with the heavy chain CDR H3 and H1 of AR3C Fab as indicated. m.c -main chain; s.c—side chain.

Similar to the AR1 antibodies, we confirmed that mAbs AR3A, AR3B, AR3C, and AR3D target overlapping but distinct epitopes on E2 (Figs 6 and S2). The four mAbs share 13 common critical amino acids with approximately half of these residues localized on the front layer of E2 (amino acids 421–452). The remaining critical residues are mostly spread throughout the Ig β sandwich and CD81 binding loop. Structurally, most of the surface-exposed residues localize towards the front of E2c on the neutralizing face of the protein (Fig 6B), in agreement with the known binding site based on the E2c-AR3C Fab crystal structure [15]. With the exception of P525, the critical residues that were identified in the original mAb characterization were also observed by flow cytometry (S2C Table). Hydrogen-bond calculations on the E2c-AR3C structure indicated that front layer residues C429, S432, and Y443 have hydrogen-bonding partners on the heavy chain of the AR3C Fab (Fig 6C). When these three residues were mutated, AR3C reactivity dropped to 1%, 55%, and 15% for these three residues, respectively, confirming their importance in the AR3C epitope. Bailey and colleagues reported that polymorphisms at four E2 amino acids (L433I, L438I, F442I, and K446E) resulted in resistance to mAb AR3C [34]. Yet in our study, we observed decreased AR3C reactivity for L438A and F442A, and enhanced AR3C binding for L433A and K446A mutants compared to wild-type (S1 Table). While side chain loss by mutation to alanine can eliminate interactions energetically important for antibody binding, in this case the differences in side chains likely also modify the conformation of E2 front layer main chain for antibody recognition.

### Effect of E2 back layer on E1E2 folding, CD81 receptor binding, and AR3 mAb reactivity

The flow cytometry data indicated that 21 individual mutations in the back layer region led to <50% binding of all nine AR1-5 antibodies (Fig 2C, S1 Table), revealing a critical role for this region in E1E2 folding. Hydrogen bond calculations suggest potential interactions between several residues in the back layer region with the central Ig scaffold and front layer, underscoring the role of E2 back layer in maintaining overall E1E2 folding (S3 Table). However, in ELISA analysis, only two mutations caused impaired global folding based on the same criteria as above (S4 Table). This observation highlights the subtle difference in antigen presentation in the different binding assays (see discussion section).

**Fig 7:**
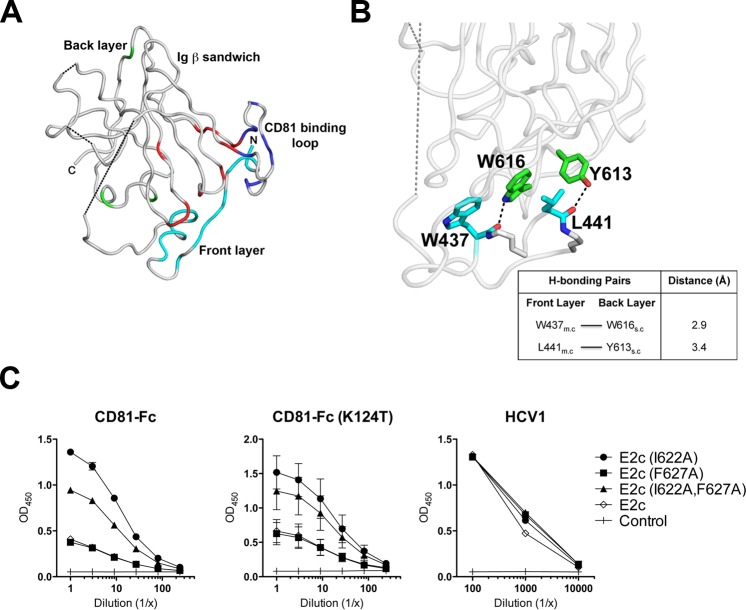
E2 back layer mutations modulate CD81 binding to the E2 front layer and CD81 binding loop. **(A)** Based on flow cytometry and ELISA analysis, and previously published results [15], residues in four distinct E2c regions were found to be important for CD81 binding: the front layer (cyan), Ig β-sandwich (red), CD81 loop (blue), and the back layer (green). The locations of mutations resulting in <25% CD81 binding in each of these regions are highlighted. **(B)** Hydrogen bond calculations indicate that two back layer residues in the α2-helix, Y613 and W616 (green), interact with L441 and W437 (cyan) of the front layer α1-helix, respectively. m.c—main chain; s.c—side chain. These interactions between front and back layer residues suggest that the back layer indirectly affects CD81 binding through structural interactions with the adjacent front layer. The residue colors follows those in Fig 1C. **(C)** Based on flow cytometry and ELISA, I622A, which was found to enhance CD81 binding, was analyzed further (along with F627A). Soluble E2c mutants harboring I622A, I622A/F627A and F627A were tested using ELISA for their ability to bind recombinant WT CD81-Fc (left panel) or a mutant that reduces CD81 dimerization, CD81-Fc (K124T) (middle panel). Binding signals to mAb HCV1 were used as an expression control for the mutants (right panel).

Recent structural data revealed that the cell surface receptor CD81 binds to the front layer and the CD81 binding loop of E2c (residues 519–535) [15]. These data confirmed results from prior mapping experiments, which proposed that several residues between 420 and 535 are important for CD81 binding [27, 37, 38]. However, prior to the crystallization of E2c, alanine scanning mutagenesis indicated that several E2 back layer residues between 613–620 might also be involved in CD81 binding [38, 39]. To evaluate these results, we screened a recombinant CD81-LEL Fc-fusion protein against the E1E2 mutant library. As expected, screening of CD81-LEL against the E1E2 mutant library confirmed that many residues within the front layer and the CD81 binding loop are required for CD81 recognition (S1 Table, Fig 7A). We also noted several residues in the central Ig β sandwich scaffold that may play an indirect role possibly through stabilization of the front layer and CD81 binding loop structures. As noted above, many back layer mutants perturbed E1E2 global folding, thus preventing us from determining whether these residues bind CD81 directly. However, we found that six back layer mutants reduced CD81 reactivity to ≤25% of wild-type reactivity without disrupting global folding: P601A, T604A, Y613A, W616A, C620A, and V633A (S1 Table). The back layer appears to affect front layer architecture indirectly with specific interactions between back layer α2 helix residues Y613 and W616 and front layer α1 helix residues W437 and L441 (Fig 7B). Y613 and W616 are within close proximity to the front layer α helix and our analysis confirms that mutation of either residue abolished CD81-LEL binding.

Conversely, I622A was found to enhance CD81-LEL binding by 56% and 49%, determined by ELISA and flow cytometry, respectively (S1 Table), while F627A did not affect binding (flow cytometry) or mildly enhanced binding by 13% (ELISA). These findings were confirmed by testing the ability of soluble E2c harboring I622A, F627A, or double mutations to bind CD81-Fc and a mutant that reduces CD81 dimerization, i.e. CD81-Fc (K124T) (Fig 7C).

Given that the E2 antigenic site 3 (AR3) is known to overlap with the CD81 binding site [15, 33], we expected the same back layer mutants that inhibited CD81 binding to also abolish AR3 antibody binding. Indeed, of the six back layer residues that are critical for CD81 binding but not overall E1E2 folding (P601, T604, Y613, W616, C620, and V633), individual mutations of four of them (P601A, T604A, Y613A, and W616A) also resulted in ≤50% reactivity to all four AR3 mAbs. Of the remaining two residues, C620A showed reduced reactivity to two AR3 mAbs (<50%), and V633A reduced binding of AR3C to <50%. Furthermore, as with CD81, I622A enhanced binding of mAbs AR3A, AR3C, and AR3D.

To characterize the back layer region further, HCV pseudoparticles (HCVpp) with individual alanine substitutions in the back layer (residues 600–645) were generated. The mutant viruses were found to be mostly poorly or non- infectious (0–27% wild-type infectivity) (S5 Table), confirming the crucial role of the back layer region. The incorporation of E1E2 onto HCVpp of 4 selected back layer mutants (W616A, I622A, V629A and R639A) was also examined (S4 Fig). While the expression of glycoproteins in transfected cell lysates was similar for wild-type and mutants (S4A Fig), E2 associated with purified HCVpp was reduced for 3 of the 4 back layer mutants (I622A, V629A and R639A) (S4B Fig), possibly caused by improper protein folding. In addition, HCVpp produced in this study were found to contain covalently linked, oligomeric forms of E2 and noncovalent E1 (S4C Fig). In a previous study of HCVpp by immunoprecipitation of transfected cell supernatant using anti-E2 mAbs, noncovalent E1E2 heterodimers, presumably E1E2 secreted from the transfected cells, were also detected [40]. In cell culture HCV (HCVcc), the majority of E1 and E2 appeared to form covalent oligomers [41].

### Residues critical for the formation of the E1E2 complex

**Fig 8:**
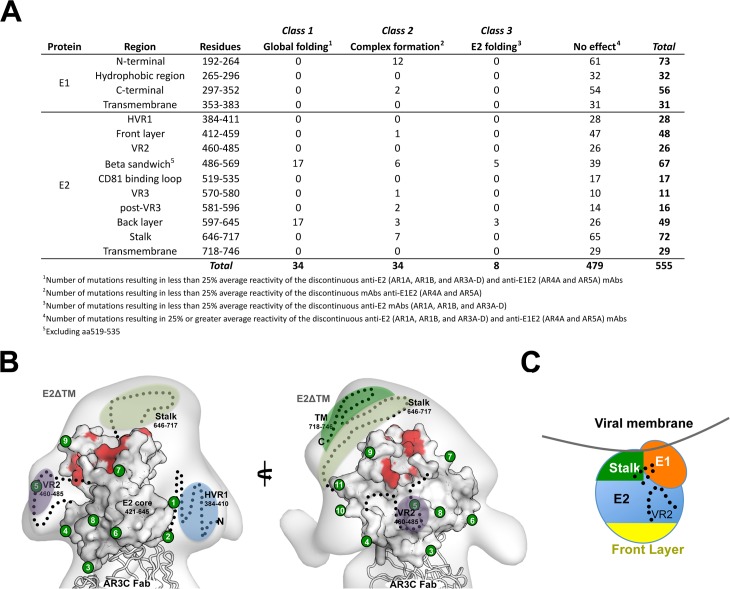
The stalk, VR2, and a glycan-free face of E2 may play a role in the E1E2 interface. **(A)** Classification of E1E2 residues based on the effects of mutations on antibody binding. **(B)** The location of **Class 2** residues (mutation affected E1E2 complex formation) are indicated in red on the structure of E2 with the transmembrane region removed (E2ΔTM) [15]. Regions that are missing or disordered in the structure are represented by black dotted lines. The area containing HVR1 is highlighted in blue, VR2 is in purple, and the stalk and transmembrane regions are in light and dark green, respectively. The locations of glycans are indicated by green circles and are numbered beginning with the N-terminus. **(C)** A general model of the E1E2 interaction indicates that the stalk region, the base of VR2, and a portion of E2 opposite of the front layer and CD81bs (i.e. VR3, post-VR3 and back layer) possibly interacts with E1. Dots indicate region of E2 that may interact with E1.

The mutant library combined with the antibody panel provided an opportunity to map the interface between E1 and E2. The panel contained mAbs that recognize discontinuous epitopes on E2 (anti-E2, i.e. AR1, AR2 and AR3), and also mAbs that recognize discontinuous epitopes requiring both E1 and E2 (anti-E1E2, i.e. AR4 and AR5). We used the two mAb types to identify potential E1E2 interface residues based on the effect of alanine substitution on their binding: (**1**) low binding by anti-E2 and anti-E1E2, (**2**) low binding by anti-E1E2 only, or (**3**) low binding by anti-E2 only. Given that antibodies in both groups recognize non-overlapping discontinuous epitopes, residues that correlate with low binding by anti-E2 and anti-E1E2 (**Class 1)** are likely to be critical to the global integrity of the E1E2 heterodimer complex. As expected from the fact that E2 can fold by itself whereas E1 folding requires E2 co-expression, all 34 **Class 1** residues were mapped to E2, to the β-sandwich and back layer (Fig 8A). On average, **Class 1** residues are 91% conserved and 6 are cysteines (S1 Table).

The 34 identified **Class 2** residues could be considered as critical residues for binding by anti-E1E2 mAbs. However, since the two anti-E1E2 antibodies, AR4A and AR5A, recognize non-overlapping epitopes, mutation of residues that severely impact binding by one mAb should not affect binding by the other. Surprisingly, the majority (31) of **Class 2** residue mutations affected binding of both mAbs in a similar manner regardless of their location on E1 or E2. This implies that these **Class 2** residues, outside the AR4A and AR5A binding epitopes, are important for formation of the E1E2 complex, either by influencing folding of E1, or by being part of the complex interface. **Class 2** residues are 97% conserved, which highlights their importance to overall structural integrity, with nine of them being cysteines. Among **Class 2** residues, 20 of 34 are located on E2 within the flanking regions of VR2 (2 cysteines), β-sandwich scaffold, VR3, post-VR3, back layer and stalk regions (Fig 8A). When visualized on the crystal structure of E2c, the **Class 2** residues strikingly cluster on one surface of E2c that is opposite to the CD81 receptor-binding site (Fig 8B). Similar to the CD81 binding site, this region is nearly glycan free, but contains flexible and disordered loops, which might require interaction with E1 to fold properly. We also tested binding of anti-E1E2 antibodies to a number of deletion mutants lacking regions harboring the **Class 2** residues (S3 Fig). Mutants lacking VR2, VR3, post-VR3 and stalk did not bind to AR4A and AR5A mAbs, in agreement with the results of the alanine scanning mutagenesis. Of note, E1E2 mutants lacking the stalk region did not bind to AR2A and AR3A mAbs suggesting that absence of stalk region can have a deleterious effect on overall E2 folding. **Class 2** residues on E1 includes 6 of the 8 E1 cysteine residues that mainly map to the nE1 region that has been crystallized and whose structure has been determined (Fig 8A, [19]).

Residues that fall under **Class 3** (low binding by anti-E2 only) are critical for E2 structural integrity but not for E1E2 complex formation. Eight such residues were mapped to either the β-sandwich (5 residues) or the back layer of E2c (3 residue). Surprisingly, **Class 3** residues are 93% conserved and none of them are cysteines.

Together, the data suggest that specific residues in the E2 near VR2, part of the glycan-free Ig β-sandwich, VR3, post-VR3, back layer and stalk appear to cluster around a region opposite to the CD81 binding site, which may interact with specific E1 residues to form the E1E2 interface. Although further investigation is required, these findings provide the basis for a general model that can describe at least some aspects of the E1E2 interface (Fig 8C).

## Discussion

Although the introduction of direct-acting antivirals (DAAs) has tremendously increased cure rates for individuals infected with HCV, the virus continues to infect between 30,000 and 100,000 people each year in the United States alone because of the lack of a prophylactic vaccine [42]. Unfortunately, high genetic variability of the virus has proven to be a major roadblock for designing a vaccine. The viral envelope glycoproteins E1 and E2 are targets of NAbs and are candidate vaccine antigens. However, the many glycosylation sites, flexible regions, and correct formation of disulfide bonds on E1E2 have hindered the production of natively folded proteins for structural studies to guide rational vaccine design.

### E2 back layer region plays a central role in E1E2 architecture

To advance our understanding of the structure and antigenicity of E1E2, we selected a panel of 13 mAbs and CD81-LEL and tested them against a complete alanine-scanning mutagenesis library of E1E2. The library was created by substituting alanine for every residue (and serine for alanine residues) of the H77 genotype 1a E1 and E2 proteins followed by high-throughput flow cytometry analysis to measure antibody reactivity to each mutant [26]. A number of mutations affected E1E2 global folding as determined by substantial reduction in reactivity to multiple conformation-dependent antibodies. Most of these residues are located in the central Ig scaffold and the back layer of E2, indicating the importance of these regions for folding of E1E2. In particular, the large number of back layer residues (42%) that impact E1E2 global folding, and the indirect effects of this region on the neighboring central Ig scaffold and distal front layer, indicate the importance of the back layer on E2 structure and function.

The high level of conservation among many back layer residues confirms the critical role of this region. Indeed, 36 of the 49 back layer residues are 90% conserved with 28 being >99% conserved (S1 Table). Our flow cytometry analysis suggested that many of the N-terminal back layer mutants perturb global E1E2 folding (Fig 2C). However, when tested by ELISA, only Y611A and R614A meet the criteria established for determining residues that impact overall protein folding (S4 Table). Such differences based on assay method were also observed for AR2A reactivity to back layer mutants (Fig 5A and 5B). AR2A is known to bind only to genotype 1a viruses unless the HVR1 region is deleted [33, 43], which raises the possibility that masking of epitopes under different conditions could affect reactivity. In ELISA, E1E2 antigens are often prepared in the form of cell lysates and the solubilized protein complex is captured by *G*. *nivalis* lectin (GNL) onto a solid support for detecting binding antibodies. In flow cytometry, E1E2 is presented as an intracellular membrane-associated antigen (S1 Table, S1 Fig). Thus, subtle differences in epitope presentation may account for variations in reactivity, predominantly in the back layer but also at other antigenic sites.

### Mutations at putative E1 fusion peptide do not disrupt E1E2 complex

While the structure and function of E1 remains elusive, the N-terminal portion (nE1) from residues 192–270 was recently crystallized and described as a disulfide-linked intertwined homodimer [19]. To our knowledge, purified E1E2 has no inter-molecular disulfide bonds [44, 45]. Thus, it remains to be determined if full-length E1 retains the above-described structure in complex with E2.

Several studies have suggested that the viral fusion peptide is located within the E1 glycoprotein [46–49]. Peptide library experiments on membrane rupture, hemifusion, and fusion suggest that the putative fusion peptide is located between residue 265–296 [48]. However, there is evidence for and against this hypothesis. The hydrophobic region spanning residues 265–296 is relatively conserved among genotypes, especially the two cysteine and two glycine residues within this region, and displays similarities to other flavivirus fusion peptides [46]. Since viral fusion peptides and fusion loops are intrinsically metastable, they are usually hidden and protected until fusion is activated [50, 51]. If this region is indeed the fusion peptide, mutations in this region should affect E1E2 assembly while maintaining CD81 receptor binding. Previous mutagenesis studies indicate that mutations within this E1 region did not affect E1E2 association or binding to CD81 receptor [46, 52]. In contrast, flow cytometry analysis of this region here showed reduced CD81 receptor binding to several mutants including Y276A (50%), G278A (50%), and D279A (48%), while E1E2 complex formation was mostly unaffected. Since receptor binding occurs in a step preceding fusion, these residues are likely affecting entry and not fusion. These conflicting results demand stronger evidence to sustain the hypothesis that the E1 region 265–296 is the fusion peptide.

### E2 CD81 binding site, antigenic region 3 and back layer region

Studies by various groups suggested that HVR1, residues 412–447, 528–535, and 612–618 are involved in CD81 reactivity by either enhancing (in the case of HVR1) or reducing CD81 binding when deleted or mutated [39, 53, 54]. Other groups have found that W420, Y527, W529, G530, and D535 were critical for CD81 binding, while H421, I422, S424, G523, T526, and F550 reduced CD81 binding by at least 50% when mutated [27, 55]. Analysis of the E1E2 library confirms that alanine mutations at the afore-mentioned 11 residues exhibit less than 25% CD81-LEL reactivity (S1 Table). In a structural context, the E2c structure and the negative-stain EM reconstruction of the E2 ectodomain bound to CD81-LEL validate the roles of the residues located at the front layer (aa 420–450) and CD81 binding loop (aa 519–535) with some residues in the β-sandwich scaffold modulating this interaction [15]. The back layer Y613 and W616 were identified as also being crucial for CD81-LEL reactivity as they exhibited less than 3% binding activity when mutated to alanine. In the E2c structure, the side chains of both residues point towards the front layer α1 helix and potentially hydrogen bond with the main chain of L441 and W437, respectively. These two residues may act as an anchor, stabilizing front layer folding and architecture of the CD81 binding site. Surprisingly, our study also found that mutation of back layer I622 enhanced CD81 binding. Recent mutagenesis studies have also corroborated the affinity enhancing effect of I622A [56].

Similarly, when several back layer residues were individually mutated, we observed reduced reactivity to AR3 mAbs, which target the front layer. Krey and colleagues suggested involvement of back layer residues 610–619 in binding of mAb HC-84 to the front layer [57]. Thus, the results presented here support the notion that the back layer can influence binding of CD81 and antibodies that target the front layer through indirect interactions. Additional interactions between back layer and β-sandwich residues were also observed, suggesting the back layer is highly involved in stabilizing the globular structure of E2.

Analysis of back layer residues using the HCVpp system shows that alanine substitution in this region is poorly tolerated, leading to severely reduced infectivity (S5 Table). Further study of four representative back layer mutants (W616A, I622A, V629A and R639A) shows that the residues can affect different aspects of E1E2 critical to the infection cycle. Reduced glycoprotein incorporation was observed in 3 of the 4 back layer mutants examined (I622A, V629A and R639A), which may partly explain the reduced viral infectivity. In contrast, W616A mutation did not affect E1E2 incorporation yet the virus was non-infectious (S4B Fig and S5 Table). These differences suggest that changes in the back layer can affect E1E2 functions in different ways (e.g. protein folding and incorporation onto virions, receptor binding, and other steps for productive infection). Although further studies are required to understand how the back layer is involved in these steps, our results underscore that this E2 region is an indispensable part of the E1E2 complex architecture.

In addition to influencing AR3 antibody binding, the E2 back layer was found to interact with mAb AR2A. Using a combination of flow cytometry and ELISA, together with previous negative-stain EM data [15], AR2A was found to recognize an antigenic site that is comprised, at least partially, of the highly conserved back layer of E2. AR2A neutralizes only some strains of HCV [33, 58], but all HCV genotypes when HVR1 is deleted, suggesting that its target residues are conserved but likely shielded by HVR1 [43, 59].

The E2 AR3, also known as the neutralizing face of E2, is devoid of N-linked glycans and overlaps with the CD81 binding site [15]. It is formed by two E2 regions, the front layer (a.a. 421–452) and the CD81 binding loop (a.a. 519–535). Although the majority of the residues in the front layer and CD81 binding loop are highly conserved (>90% conservation), natural variations are observed at a number of hot spots (<75% conservation) that include S424 (16%), E431 (11%), N434 (41%), W437 (46%), L438 (43%), G440 (56%), Q444 (0.2%), H445 (54%), K446 (65%), R521 (65%), S522 (33%), A524 (47%), S528 (25%), A531 (12%) and D533 (33%) (S1 Table). Among the conserved residues, alanine substitution at T425, L427, N428, C429, G436 and L441 in the front layer region, T519, D520, G523, W529, G530 and D535 in the CD81 binding loop, greatly reduced the binding of the four AR3-specific mAbs. Obviously, viruses with natural variations at these conserved positions, provided not defective in replication, can potentially escape the AR3 mAbs. Nevertheless, the natural variations L442 in the genotype 5 isolates SA13 and UKN5.15.7, N519 in the genotype 2 isolate UKN2a1.2, and E535 in genotype 3 isolates S52 and UKN3a1.28, have not rendered the viruses more sensitive or resistant to mAb AR3A [35, 60, 61]. On the other hand, natural variations at the conserved L433 (94%), and the less conserved L438 (43%), F442 (81%) and K446 (65%), and E431 and a set of 3 residues outside AR3 (V538, L546 and V563) in some genotype 1 isolates, have been reported to confer resistance to some AR3 mAbs [34]. Interestingly, alanine substitution at L433, L438, F442 and K446 had variable effects on the binding of AR3 mAbs (S1 Table). Given the differences in size, polarity, and charge, it is notable that alanine mutations at L433 and K466 have the opposite effect on AR3C binding compared to naturally occurring mutations to isoleucine, histidine, glutamic acid, or asparagine, which have been observed in E1E2 sequences [34].

Our analysis also shows that C429 is critical for binding of all four AR3 mAbs, suggesting that this amino acid is a crucial contact point for the mAbs. It is likely that the C429-C503 disulfide bond is critical for the integrity of the E2 CD81 binding site, since mutation of either specifically affects binding by AR3 antibodies and CD81-LEL, but not the other mAbs to discontinuous epitopes (S1 Table, [56]).

Overall, these data indicate that AR3 antibodies recognize overlapping but distinct epitopes targeting the front layer region and the CD81 binding loop of E2c, and that the surrounding region including the Ig central scaffold may play an indirect role in mAb binding.

### Putative E1E2 interface

MAbs AR4A and AR5A recognize the quaternary structure of E1E2 complex and cross-neutralize multiple HCV genotypes [34, 35]. Screening the mAbs against the E1E2 mutant library revealed that 15 residues in E1 and 21 residues in E2 are required for binding of mAbs AR4A or AR5A (S1 Table). In fact, among the 34 mutants (**Class 2** residues, Fig 8A), flow cytometry analysis revealed that only 4 exhibited <25% binding for AR4A or AR5A alone (two E1 and two E2 residues). Previous studies have revealed minimal overlap between the AR4A and AR5A epitopes suggesting that the remaining 30 residues, that reduce binding by both AR4A and AR5A, are required for E1E2 complex formation [35]. Among these 30 residues, 18 E2 residues cluster at the junction of the non-neutralizing and occluded faces of E2c, in an area with few glycosylation sites (Fig 8B).

The three E1 residues (I308, A330, and M345) recently hypothesized by Douam and coworkers to be involved in functional E1E2 heterodimerization and viral fusion [13] did not affect AR4A or AR5A binding as determined by flow cytometry. The same group also proposed that the E2 region spanning amino acids 581–650 could be involved in a “crosstalk” with E1 and plays an important role for E1E2 function [13]. This region encompasses a flexible area and the back layer of E2c, and contains five residues found to be critical for AR4A and/or AR5A reactivity. Overall, results from AR4A and AR5A mapping support the hypothesis that some residues between E2 581–650 are involved in interactions with E1.

The variable regions of E2—HVR1, VR2, and VR3—have been shown to be unnecessary for folding of soluble, recombinant E2 since conformational mAbs retain their ability to bind even with one, two, or all three variable regions deleted [15, 62]. Yet, in the context of virion-incorporated glycoproteins, VR2 and VR3 (also known as IgVR, intergenotypic variable region) appear to affect E2 folding, assembly of E1E2 complex, receptor binding, HCVpp entry, and HCVcc infectivity, highlighting differences in requirements for the variable regions between recombinant E2 and virion-associated E1E2 [63]. We found that mutations within the variable regions of E2 were generally well tolerated since binding of CD81-LEL, and mAbs with continuous and discontinuous epitopes, was maintained for most mutants. However, mutations in 9 residues, F465A, Q467A, I472A in VR2, G573A, C581A, D584A, C585A in VR3, F586A and Y594A in post-VR3, reduced binding of E1E2-specific mAbs AR4A and AR5A by at least 50% compared to wild-type E1E2 without affecting binding of other mAbs. Thus, the variable regions seem to play a functional role in the formation of E1E2 complex.

Six of the 14 **Class 2** E1 residues are cysteines (C207, C226, C229, C238, C304 and C306), suggesting a role for disulfide bridges in E1 folding/stabilization and/or E1E2 complex formation. Due to limitation of the antibody reagents and the lack of properly folded E1 antigen, we cannot distinguish E1 residues important for E1 folding versus E1E2 complex formation.

Of these 6 cysteines in E1, four are located in the N-terminus and implicated in inter- and intra-molecular disulfide bonds [19]. Seven of the 20 important E2 residues that affect AR4A and AR5A binding are cysteines (C459, C486, C569, C585, C597, C652 and C677). Mutation of these cysteines did not affect the binding of other conformation-dependent mAbs, suggesting that they may not affect the overall fold of E2, but may instead play a role in maintaining the E1E2 complex. Alternatively, these cysteine residues could instead be unpaired and required for HCV infectivity. Fraser *et al*. proposed that both HCVpp and HCVcc systems require free thiol groups for entry and that E1E2 undergoes a shift from a reduced to an oxidized state during receptor attachment [64]. Moreover, McCaffrey and colleagues demonstrated that E2 can tolerate the presence of several free cysteines [65]. Thus, free cysteines may indirectly affect the binding of AR4A and AR5A while not impacting overall protein conformation. Altogether, our data suggest that a glycan-free face of E2, distal from the front layer, interacts with E1 forming the complex interface. It also appears that residues within the post-VR3 region, back layer and the E2 stalk may play a role in complex formation, either directly or indirectly (Fig 8C). The importance of variable regions and E2 stalk in E1E2 assembly was also confirmed independently using mutants that lacked these regions (S3 Fig).

Of note, it is yet to be proven that the Class 2 residues are physically in contact to form the E1E2 interface. Our attempts to study this by immunoprecipitation have not yielded conclusive results of a complete disruption of E1E2 complex formation. The transmembrane regions of E1 and E2 are crucial for E1E2 dimerization and specific mutations within either domain can reduce up to 75% of heterodimer formation [12]. It is possible that the interactions between E1 and E2 ectodomains are relatively feeble to allow for conformational rearrangement during viral entry and may not be easily detected in the presence of the transmembrane regions.

## Conclusion

Overall, through the use of conventional ELISA and high-throughput flow cytometry analysis, we screened CD81-LEL and a panel of mAbs targeting five antigenic regions of E1E2 against a comprehensive alanine mutant library, encompassing the entire E1E2 protein sequence of the prototypic genotype 1a H77 strain. This approach offers a global perspective of folding and expression of the complex, and provides insight into E1E2 structure and antigenicity. The results reported here are in agreement with the previously mapped targets for HCV1, AP33, and mAbs specific for AR1, AR2, and AR3. Residues in the E2 back layer appear to play a central role in maintaining not only E2 structure through interactions with the Ig scaffold and front layer, but also in overall folding of the E1E2 complex. Importantly, residues located on the back layer of E2 were also found to modulate AR3 and CD81-LEL binding, likely by stabilizing the structure of the front layer and CD81 binding loop. The E2 back layer region also appears to be central to E2 folding and function because individual alanine substitutions in this region universally reduced viral infectivity. It is evident that protein-engineering efforts should consider the contribution of several residues in the back layer region and the potential for global misfolding as a consequence of their mutation. This study also provides preliminary evidence of the location of the E1E2 interface at a glycan-deficient region opposite of the CD81 binding site. Several flexible or disordered loops present in this region have the potential to interact with E1 to form the functional E1E2 complex. Further studies to better define the E1E2 interface, particularly in the isolation and mapping of mAbs to E1 discontinuous epitopes and to study the mutations in the context of authentic virus, will greatly facilitate our understanding of the E1E2 complex. Intergenotypic incompatibilities between E1 and E2 suggesting coevolution of glycoproteins within a genotype should also be considered while studying complex formation [66].

In the absence of complete E1 and E2 structures, it is difficult to fully understanding how each alanine substitution influences E1 and E2 folding. The data here are useful for identifying key residues for antibody binding and distal protein regions required to form the epitopes. However, the data do not have the power to predict the effect of substituting E1E2 residues with amino acids of diverse chemical properties as seen in natural viral sequences. Other approaches, such as selection of antibody escape virus mutations or deep mutational scanning analysis, will complement alanine scanning in the study of E1E2 complex.

This set of complete alanine scanning mutagenesis data will be valuable to inform design of new E1E2 constructs with improved biochemical properties (e.g. folding, solubility and stability) for structural studies and immunization. We believe that a folded, soluble complex of E1E2 ectodomain will be highly valuable to the field. Our study has established the importance of E2 VR2 flanking regions, VR3, post-VR3 and back layer regions in E1E2 formation and these regions should be taken into account for future protein engineering and vaccine design efforts. Future research on E1E2 interface could lead to improved immunogen engineering for vaccine design.

Our discussion of this large dataset has been restricted here to the antigenic regions, CD81 binding, and E1E2 complex formation because of publication length considerations. However, the entire dataset compiling results from the ELISA and flow cytometry analysis, amino-acid conservation, and previously published mutagenesis data are available online as an Excel spreadsheet (S1 Table) and we welcome further interpretation and discussion of the data.

## Materials and methods

### HCV E1E2 mutant library construction

Comprehensive high-throughput alanine scanning mutagenesis was carried out on an HCV E1E2 expression construct (genotype 1a, strain H77; reference sequence NC_004102) encoding a C-terminal V5 epitope tag. Individual residues of E1 and E2 were mutated to alanine while existing alanine residues were mutated to serine to create a library of clones, each with a single point mutation. Overall, 545 mutants were generated by Integral Molecular, Inc., covering 98.2% of the E1E2 target residues. The sequence of each clone was confirmed by DNA sequencing (Macrogen) and the library clones were arrayed in 384-well format with each well containing one mutation [26]. Remaining constructs were found to have additional mutations and to complete the library, these 10 alanine mutations (R237A, C272A, Q336A, D346A, T396A, C452A, K562A, Y613A, Y624A, and W712A) were introduced into the H77C E1E2 sequence [67] using the QuikChange Lightening Site-Directed Mutagenesis kit (Stratagene) and PCR primers for each mutation (Integrated DNA Technologies). The sequence of these clones was confirmed by DNA sequencing (Retrogen).

### Anti-HCV antibody panel

Mouse mAb A4 and human mAb IGH526 target the N-terminal (residues 197–207) and C-terminal portion (residues 313–327, linear component of IGH526) of E1, respectively [23, 28, 29, 40]. mAbs HCV1 and AP33 recognize E2 residues 412–423, a region that is known for inducing potent, cross-reactive NAbs [30–32] (reviewed in [20]). The antibodies A4, IGH526, HCV1, and AP33 have been described previously and were produced in-house as recombinant antibodies [23, 28–32, 40].

The AR1-5 antibodies were isolated previously from an HCV antibody library by phage display [33, 35]. They recognize three distinct E2 antigenic regions (AR1-3) and two E1E2 antigenic regions (AR4-5). mAbs recognizing AR1 are strain-specific and mostly non- or weakly neutralizing, suggesting this region is occluded in native virions [33]. On the other hand, mAbs targeting AR2 and AR3 are capable of neutralizing several viral genotypes. AR3 mAbs recognize the neutralizing face of E2c, which overlaps with the CD81 binding site while mAb AR2A binds to the back layer of the E2 protein [15, 33]. AR4 and AR5 mAbs are specific for the E1E2 complex and recognize non-overlapping epitopes. mAb AR4A has been shown to cross-neutralize the six major HCV genotypes and protected against the human liver chimeric mouse model from HCV challenge [35, 60].

### Overview of methodology

The flow cytometry method measures binding of antibodies to intracellular, membrane-associated E1E2. In ELISA, antigens are typically presented as solubilized E1E2 in transfected cell lysate, enriched onto the microwell surface. Although extraction of E1E2 by non-denaturing detergents and enrichment of antigens by lectin capture could potentially alter epitope presentation, the antigenicity of the two different forms of E1E2 antigens are mostly equivalent with some exceptions, predominantly in the E2 back layer. We also confirmed that the antigenicity of cell lysate-derived E1E2 remains relatively stable over time, even after several freeze-thaw cycles.

### Immunofluorescence assays

For the mutations introduced by Integral Molecular, Inc., the HCV E1E2 mutant library, arrayed in 384-well microplates, was transfected into HEK-293T cells (ATCC CRL-11268) and allowed to express for 22 hours. The cells were washed in PBS supplemented with calcium and magnesium, fixed in 4% paraformaldehyde (Electron Microscopy Sciences), and permeabilized with 0.1% (wt/vol) saponin (Sigma-Aldrich) in PBS supplemented with calcium and magnesium. Cells were stained with mAbs (0.33 to 2.0 μg/ml) diluted in 10% normal goat serum (NGS) (Sigma), 0.1% w/v saponin, pH 9.0. Optimal mAb concentrations and binding conditions were determined using an independent immunofluoresence curve against wild-type E1E2 for each mAb. A concentration within the linear range and with suitable signal to background ratio (>5) was chosen for the library screening. The cells were incubated with anti-HCV antibody for 1 hour at 20°C, or overnight at 4°C, followed by washing three times with supplemented PBS and 0.1% saponin and a subsequent 30-minute incubation with Alexa Fluor 488-conjugated secondary antibody (Jackson ImmunoResearch) in 10% NGS, 0.1% saponin, and supplemented PBS. Stained cells were washed three times with supplemented PBS and 0.1% saponin, twice with PBS without calcium or magnesium, and were re-suspended in Cellstripper (Cellgro) plus 0.1% BSA (Sigma-Aldrich). Cellular fluorescence was detected using the Intellicyt high throughput flow cytometer (HTFC, Intellicyt). Background fluorescence was determined by fluorescence measurement of vector-transfected control cells. mAb reactivity against each mutant HCV E1E2 clone was calculated relative to wild-type E1E2 by subtracting the signal from mock-transfected controls and normalizing to the signal from wild-type HCV E1E2-transfected controls.

The reactivity of the mAb panel to the Q336A, D346A, T396A, C452A, K562A, Y613A, Y624A, and W712A mutants was measured essentially as described above, but 0.5% saponin (Sigma-Aldrich) was used. As above, a titration curve for mAb binding to wild-type E1E2 was performed to determine the optimal mAb concentration (linear range). Fluorescence was detected using a LSR II cytometer (BD Biosciences). Reactivity was normalized to wild-type with background binding removed.

### Epitope mapping

Mutated residues within critical clones were identified as critical to the mAb epitope if they did not support reactivity of the test mAb but did support reactivity of other control anti-HCV mAbs. V5-tag expression was also measured to assess the effect of each mutation on overall E1E2 expression in transfected cells. The counter-screen strategy and V5 expression tests facilitates the exclusion of E1E2 mutants that are locally misfolded or that have an expression defect [68, 69]. To be highlighted as an important residue, binding thresholds were established in which there was <25% binding of the mAb of interest but >75% binding of appropriate continuous and discontinuous control mAbs. Mutations resulting in <50% binding for all discontinuous mAbs were flagged as causing perturbations in global E1E2 folding.

### ELISA

#### (i) Alanine mutants

Specific E1E2 residues selected based on conservation across genotypes or by region (eg. back layer) were individually mutated to alanine using the QuikChange Lightening Site-Directed Mutagenesis kit (Stratagene) and specific PCR primers (Integrated DNA Technologies). ELISA was used to assess the binding capability of the mAbs (or CD81-LEL) to the E1E2 mutants [35]. Briefly, cell lysate from transfected HEK-293T cells containing mutant E1E2 antigen was captured on ELISA plates pre-coated by *G*. *nivalis* lectin (GNL, 5 μg/mL) and blocked with nonfat milk (4% wt/vol, BioRad) in PBS/0.05% Tween20. Clarified cell lysate containing the mutant antigen was used at a 1:5 dilution. After washing, mAb or CD81-LEL was added to the plates at a concentration of 1 μg/mL and binding was measured using an HRP-conjugated IgG antibody and TMB substrate (Pierce). Non-transfected cell lysate was used as a negative control to determine background signal for each mAb. Binding signals to the E1E2 alanine mutants were then normalized to binding to wild-type E1E2. Binding studies for each mutant were repeated at least two times.

#### (ii) E2c construct

Soluble E2c constructs expressing WT E2c [15] or E2c with alanine mutations (generated using QuikChange Lightning Site-Directed Mutagenesis Kit from Agilent) in the back layer (I622A, F267A and double mutant), were transfected into HEK-293T cells in the presence of kifunensine as described above. Dilutions of cell supernatant containing soluble E2c were assessed for ability to bind WT CD81-Fc and CD81-Fc (K124T), a mutant that reduces dimerization of CD81 [70] using ELISA as described above. Supernatants from cells transfected with pAdvantage plasmid (without E2c) was used as a negative control. Diluted supernatants from WT E2c and back layer mutants were also assessed for expression to bind mAb HCV1 (different range of dilutions used due to high sensitivity of HCV1 antibody).

#### (iii) Deletion mutants

Different versions of E1E2 were created using the full-length H77 E1E2 construct [67] to remove regions of E2 (-HVR1, -VR2, -VR3, -post-VR3 and -stalk). The constructs were expressed in HEK-293T cells by co-transfection of expression plasmid with pAdvantage (Promega) and transfection agent polyethylenimine (PEI; Polysciences) as described previously [71]. The binding capability of mAbs to E1E2 was assessed by ELISA as indicated above. Briefly, E1E2 constructs expressed in HEK-293T cells were captured from cell lysates (undiluted) onto microwells pre-coated with *G*. *nivalis* lectin (GNL), AR4A or AR5A mAbs (5 μg/ml) as indicated. After blocking and washing (same as above section), captured E1E2s were probed by biotinylated mAbs A4, HCV1, AR2A, AR3A, AR4A and AR5A. Binding was measured using HRP- Streptavidin (Jackson ImmunoResearch Laboratories, Inc.). Lysates from cells transfected with H77 E1E2 (full length) was used as positive control while those transfected with pAdvantage plasmid alone (without E1E2) was used as a negative control.

### HCV E1E2 infectivity assay (Integral Molecular)

Lentiviral reporter viruses pseudotyped with HCV E1E2 (HCVpp) were produced essentially as described [72, 73] but in 384-well plates, by co-transfecting the individual expression plasmids of wildtype and mutant E1E2 with a plasmid encoding HIV core (gag-pol [74]) and luciferase (pNL4-3.lucR−E−) [75]. Cells were incubated at 37°C in 5% CO_2_ to allow for transfection and pseudovirus production. Supernatants were harvested 48 to 72 hours post-transfection and diluted 1:1 with 16 μg/ml Dextran/DMEM and stored at -80°C. Target Huh-7 cells were plated at 0.8 x 10^6^ cells/well in DMEM containing additives and incubated at 37°C in 5% CO_2_ overnight. The following day, virus harvests were thawed, medium was removed from the cells and 40 μl virus was added, cells were then incubated at 37°C. At 24 hours post-infection, 100 μl of fresh media was added to each well. Infected target cells were lysed 72 hours post-infection and lysates were assayed for luciferase activity (Promega). The raw luciferase activities for mutants were background-subtracted and then normalized to the average values obtained for wild-type E1E2.

### Analysis of E1 and E2 in the context of HCVpp

HCVpp was generated by cotransfection of 293T cells with pNL4-3.lucR−E− and the corresponding expression plasmid encoding wildtype or mutant E1E2 genes as described previously [35]. Cell lysates and culture supernatants were harvested 72 hours post-transfection for immunoblotting, infectivity assay and purification of virions. HCVpp was pelleted by centrifugation of culture supernatant at 16,000 rpm for 1 hour, resuspended, and purified over a 20% (wt/vol) sucrose cushion [35, 76]. Envelope glycoproteins E1 and E2 were detected by immunoblotting using biotinylated mouse mAb A4 [28] and mAb HCV1 [24] and the IRDye680RD Streptavidin (1:2,000) and IRDye800CW goat anti-human IgG secondary antibodies (1:10,000) (LI-COR Biosciences), respectively. HIV-1-p24 was detected using biotinylated mouse monoclonal antibody (diluted to 1:1,000; Aalto Bio Reagents). The immunoblots were analyzed with the Odyssey Infrared Imaging System and Image Studio software (LI-COR Biosciences).

### E1 and E2 amino acid conservation and hydrogen bonding analysis

For amino-acid conservation, data were obtained from the NIAID Virus Pathogen Database and Analysis Resource (ViPR) online through the web site at http://www.viprbrc.org [77]. 2,345 complete genomes encompassing genotypes 1 through 7 as well as 52 unclassified genomes were preliminarily identified for HCV. Further inclusion criteria included: human host only, genders, all geographical regions, and no defined collection period or sample source (e.g. serum, plasma, etc.). The criteria narrowed the results to 902 E1 and E2 sequences that were analyzed for sequence variation (SNP) at the amino-acid level. Percent conservation was calculated by dividing the total number of sequences analyzed by the number of sequences exhibiting the H77 reference strain (NC_004102) amino acid and subsequently converting to a percentage. Hydrogen bond calculations were performed using LigPlot [78]. Complete calculation results for the E2c—AR3C PDB file 4MWF are available upon request.

## Supporting information

S1 Fig Presentation of E1E2 for flow cytometry and ELISA. Shotgun mutagenesis followed by whole cell analysis using flow cytometry offers an alternative to ELISA. **(A)** High-throughput shotgun mutagenesis was used to create a plasmid mutant library for the entirety of E1E2, with each clone having a defined alanine mutation. Prior to screening the mutant library, each primary mAb was tested against wild-type E1E2 to determine optimal mAb concentration and conditions to achieve high signal to background ratio. HEK-293T cells were transfected with individual mutants from the library and subsequently fixed and permeabilized. Each mAb was tested against each mutant in the library by flow cytometry using a fluorescent secondary mAb to detect immunoreactivity. Background signal from mock-transfected cells was subtracted and the signals were normalized to the wild-type E1E2 positive control [26]. **(B)** Site-directed mutagenesis was performed on individual conserved residues of E1E2. Mutant E1E2-containing plasmids were transfected into HEK-293T cells and the cell lysate was added to lectin-coated ELISA plates at either high (1:5) or low (1:50) concentration. The panel of mAbs was tested against the mutant cell lysate using an HRP-conjugated secondary antibody and TMB substrate to detect reactivity. Background signal from mock-transfected cell lysate was subtracted and the signal was normalized to the wild-type E1E2 positive control.
(TIF)

S2 Fig Critical residues for AR3 antibodies. Data shown are the mutations resulting in ≤25% mean reactivity, expressed as percent of wild-type AR3B-D mAbs, but >75% of at least one control mAb, using the E1E2 mutant library and flow cytometry analysis (see Fig 6A for AR3A data). Mutants resulting in poor E1E2 expression (<40%) as determined by the C-terminal V5 tag and those implicated in global misfolding were removed. Binding assays were performed twice with the range indicated.
(TIF)

S3 Fig Variable regions and stalk of E2 are critical for assembly of E1E2 complex. The importance of variable regions (HVR1, R2, VR3, post-VR3) and stalk of E2 for E1E2 complex formation was tested by ELISA. E1E2 from HEK-293T cell lysates (undiluted) co-transfected with deletion mutants lacking these regions and pAdv plasmid was captured using GNL, AR4A or AR5A (as indicated) and evaluated for binding using a panel of antibodies. Full-length E1E2 without deletions (E1E2) and pAdv plasmid (Control) were used as positive and negative control, respectively.
(TIF)

S4 Fig Functional analysis of E2 back layer. HCVpp was generated by transfecting 293T cells with plasmids expressing wild-type or back layer E1E2 mutant (W616A, I622A, V629A and R639A). **A**) Transfected cell lysates were analyzed by reducing SDS-PAGE and immunoblotting using biotinylated mAb A4 for E1 (red) and mAb HCV1 for E2 (green). E1 and E2 in mutants were quantified as a percentage of wild-type levels (middle and right panels). **B**) Purified virions were analyzed by reducing SDS-PAGE and immunoblotting using mAb HCV1 for E2 (green) and anti-p24 for HIV-1-p24 (red). Percentage of E2 (relative to wild-type) was normalized to corresponding p24 levels (right panel). **C**) Purified virions were analyzed by non-reducing (left panel) and reducing (right panel) SDS-PAGE and immunoblotting using biotinylated mAb A4 for E1 (red) and mAb HCV1 for E2 (green). M, Molecular Marker; WT, wild-type. Experiments were performed in duplicate.
(TIF)

S1 Table Percent reactivity of the mAb panel and CD81-LEL to E1E2 mutants. Mean reactivity determined by flow cytometry (FC) and ELISA, if available, is expressed as % wild-type binding level. Results from previous mapping experiments by others and those from our study are included for comparison. The percent conservation of each residue is included. Flow cytometry and ELISA analyses were performed as described in materials and methods.
(XLSX)

S2 Table Comparison of flow cytometry data with previous mapping experiments. Our present flow cytometry (FC) and ELISA data with previously published ELISA data [35] were compared for mAbs AR1A & AR1B **(A)**, AR2A **(B)**, and AR3A-D **(C)**. Coloring is based on percent reactivity relative to wild-type: <25%, red; 25–50%, orange; 51–75%, green; >75%, white; and—, not tested. *likely perturbs global folding based on control mAb reactivity by flow cytometry.
(XLSX)

S3 Table Back layer hydrogen bonding partners. Hydrogen bond interactions between several residues in the back layer region (BLR) and the front layer (FLR), the central Ig scaffold or the variable region 3 (VR3). Data were calculated from the E2 structure, PDB ID 4MWF [15]. m.c—main chain; s.c—side chain.
(XLSX)

S4 Table E2 back layer mutations modulate CD81 binding to the E2 front layer and CD81 binding loop. The percent reactivity (given as percent of wild-type E1E2) of a panel of mAbs along with CD81-LEL, was determined for several back layer alanine mutants using either ELISA or flow cytometry (FC). Coloring corresponds to the level of binding: ≤25%, red; 26–50%, orange; 51–75%, green; 76–150%, white; and >150%, blue. Reactivity shown is a mean of 2–3 experiments. *Based on FC, these mutants may inhibit proper global folding of E1E2. ^#^A—>G mutant tested by ELISA; A—>S mutant tested by FC. —, not tested.
(XLSX)

S5 Table Effect of back layer mutation on HCVpp infectivity. A panel of HCVpp with point mutation in the E2 back layer (residues 600–645) were generated. Infectivity of each mutant was measured in triplicate using Huh-7 cells and the results are shown as a percentage of infectivity relative to wild-type HCVpp.
(XLSX)

## References

[1] Fauvelle C, Lepiller Q, Felmlee DJ, Fofana I, Habersetzer F, Stoll-Keller F, et al (2013) Hepatitis C virus vaccines—progress and perspectives. Microb Pathog 58:66–72. doi: 10.1016/j.micpath.2013.02.005 23499591 10.1016/j.micpath.2013.02.005

[2] Halliday J, Klenerman P, Barnes E. (2011) Vaccination for hepatitis C virus: closing in on an evasive target. Expert Rev Vaccines 10:659–72. doi: 10.1586/erv.11.55 21604986 10.1586/erv.11.55 PMC3112461

[3] El-Serag HB, Rudolph KL. (2007) Hepatocellular carcinoma: epidemiology and molecular carcinogenesis. Gastroenterology 132:2557–76. doi: 10.1053/j.gastro.2007.04.061 17570226 10.1053/j.gastro.2007.04.061

[4] Lindenbach BD, Rice CM. (2013) The ins and outs of hepatitis C virus entry and assembly. Nat Rev Microbiol 11:688–700. doi: 10.1038/nrmicro3098 24018384 10.1038/nrmicro3098 PMC3897199

[5] de Martel C, Maucort-Boulch D, Plummer M, Franceschi S. (2015) World-wide relative contribution of hepatitis B and C viruses in hepatocellular carcinoma. Hepatology 62:1190–200. doi: 10.1002/hep.27969 26146815 10.1002/hep.27969 PMC5019261

[6] Hoofnagle JH, Sherker AH. (2014) Therapy for hepatitis C—the costs of success. N Engl J Med 370:1552–3. doi: 10.1056/NEJMe1401508 24725236 10.1056/NEJMe1401508

[7] Pawlotsky JM. (2014) New hepatitis C therapies: the toolbox, strategies, and challenges. Gastroenterology 146:1176–92. doi: 10.1053/j.gastro.2014.03.003 24631495 10.1053/j.gastro.2014.03.003

[8] Zeisel MB, Felmlee DJ, Baumert TF. (2013) Hepatitis C virus entry. Curr Top Microbiol Immunol 369:87–112. doi: 10.1007/978-3-642-27340-7_4 23463198 10.1007/978-3-642-27340-7_4

[9] Feneant L, Levy S, Cocquerel L. (2014) CD81 and hepatitis C virus (HCV) infection. Viruses 6:535–72. doi: 10.3390/v6020535 24509809 10.3390/v6020535 PMC3939471

[10] Khan AG, Miller MT, Marcotrigiano J. (2015) HCV glycoprotein structures: what to expect from the unexpected. Curr Opin Virol 12:53–8. doi: 10.1016/j.coviro.2015.02.004 25790756 10.1016/j.coviro.2015.02.004 PMC4505365

[11] Li Y, Modis Y. (2014) A novel membrane fusion protein family in Flaviviridae? Trends Microbiol 22:176–82. doi: 10.1016/j.tim.2014.01.008 24569295 10.1016/j.tim.2014.01.008 PMC3985287

[12] Ciczora Y, Callens N, Penin F, Pecheur EI, Dubuisson J. (2007) Transmembrane domains of hepatitis C virus envelope glycoproteins: residues involved in E1E2 heterodimerization and involvement of these domains in virus entry. J Virol 81:2372–81. doi: 10.1128/JVI.02198-06 17166909 10.1128/JVI.02198-06 PMC1865936

[13] Douam F, Dao Thi VL, Maurin G, Fresquet J, Mompelat D, Zeisel MB, et al (2014) Critical interaction between E1 and E2 glycoproteins determines binding and fusion properties of hepatitis C virus during cell entry. Hepatology 59:776–88. doi: 10.1002/hep.26733 24038151 10.1002/hep.26733

[14] Krey T, d'Alayer J, Kikuti CM, Saulnier A, Damier-Piolle L, Petitpas I, et al (2010) The disulfide bonds in glycoprotein E2 of hepatitis C virus reveal the tertiary organization of the molecule. PLoS Pathog 6:e1000762 doi: 10.1371/journal.ppat.1000762 20174556 10.1371/journal.ppat.1000762 PMC2824758

[15] Kong L, Giang E, Nieusma T, Kadam RU, Cogburn KE, Hua Y, et al (2013) Hepatitis C virus E2 envelope glycoprotein core structure. Science 342:1090–4. doi: 10.1126/science.1243876 24288331 10.1126/science.1243876 PMC3954638

[16] Khan AG, Whidby J, Miller MT, Scarborough H, Zatorski AV, Cygan A, et al (2014) Structure of the core ectodomain of the hepatitis C virus envelope glycoprotein 2. Nature 509:381–4. doi: 10.1038/nature13117 24553139 10.1038/nature13117 PMC4126800

[17] Modis Y. (2013) Class II fusion proteins. Adv Exp Med Biol 790:150–66. doi: 10.1007/978-1-4614-7651-1_8 23884590 10.1007/978-1-4614-7651-1_8 PMC7123093

[18] Kong L, Lee DE, Kadam RU, Liu T, Giang E, Nieusma T, et al (2016) Structural flexibility at a major conserved antibody target on hepatitis C virus E2 antigen. Proc Natl Acad Sci U S A 113:12768-73. doi: 10.1073/pnas.1609780113 . 27791120 10.1073/pnas.1609780113 PMC5111675

[19] El Omari K, Iourin O, Kadlec J, Sutton G, Harlos K, Grimes JM, et al (2014) Unexpected structure for the N-terminal domain of hepatitis C virus envelope glycoprotein E1. Nat Commun 5:4874 doi: 10.1038/ncomms5874 25224686 10.1038/ncomms5874 PMC4175578

[20] Ball JK, Tarr AW, McKeating JA. (2014) The past, present and future of neutralizing antibodies for hepatitis C virus. Antiviral Res 105:100–11. doi: 10.1016/j.antiviral.2014.02.013 24583033 10.1016/j.antiviral.2014.02.013 PMC4034163

[21] Wang Y, Keck ZY, Foung SK. (2011) Neutralizing antibody response to hepatitis C virus. Viruses 3:2127–45. doi: 10.3390/v3112127 22163337 10.3390/v3112127 PMC3230844

[22] Kong L, Jackson KN, Wilson IA, Law M. (2015) Capitalizing on knowledge of hepatitis C virus neutralizing epitopes for rational vaccine design. Curr Opin Virol 11:148–57. doi: 10.1016/j.coviro.2015.04.001 25932568 10.1016/j.coviro.2015.04.001 PMC4507806

[23] Kong L, Kadam RU, Giang E, Ruwona TB, Nieusma T, Culhane JC, et al (2015) Structure of hepatitis C virus envelope glycoprotein E1 antigenic site 314–324 in complex with antibody IGH526. J Mol Biol 427:2617–28. doi: 10.1016/j.jmb.2015.06.012 26135247 10.1016/j.jmb.2015.06.012 PMC4523428

[24] Kong L, Giang E, Robbins JB, Stanfield RL, Burton DR, Wilson IA, et al (2012) Structural basis of hepatitis C virus neutralization by broadly neutralizing antibody HCV1. Proc Natl Acad Sci U S A 109:9499–504. doi: 10.1073/pnas.1202924109 22623528 10.1073/pnas.1202924109 PMC3386053

[25] Kong L, Giang E, Nieusma T, Robbins JB, Deller MC, Stanfield RL, et al (2012) Structure of hepatitis C virus envelope glycoprotein E2 antigenic site 412 to 423 in complex with antibody AP33. J Virol 86:13085–8. doi: 10.1128/JVI.01939-12 22973046 10.1128/JVI.01939-12 PMC3497658

[26] Davidson E, Doranz BJ. (2014) A high-throughput shotgun mutagenesis approach to mapping B-cell antibody epitopes. Immunology 143:13–20. doi: 10.1111/imm.12323 24854488 10.1111/imm.12323 PMC4137951

[27] Owsianka AM, Timms JM, Tarr AW, Brown RJ, Hickling TP, Szwejk A, et al (2006) Identification of conserved residues in the E2 envelope glycoprotein of the hepatitis C virus that are critical for CD81 binding. J Virol 80:8695–704. doi: 10.1128/JVI.00271-06 16912317 10.1128/JVI.00271-06 PMC1563869

[28] Dubuisson J, Hsu HH, Cheung RC, Greenberg HB, Russell DG, Rice CM. (1994) Formation and intracellular localization of hepatitis C virus envelope glycoprotein complexes expressed by recombinant vaccinia and Sindbis viruses. J Virol 68:6147–60. 8083956 10.1128/jvi.68.10.6147-6160.1994 PMC237034

[29] Meunier JC, Russell RS, Goossens V, Priem S, Walter H, Depla E, et al (2008) Isolation and characterization of broadly neutralizing human monoclonal antibodies to the e1 glycoprotein of hepatitis C virus. J Virol 82:966–73. doi: 10.1128/JVI.01872-07 17977972 10.1128/JVI.01872-07 PMC2224608

[30] Broering TJ, Garrity KA, Boatright NK, Sloan SE, Sandor F, Thomas WD Jr., et al (2009) Identification and characterization of broadly neutralizing human monoclonal antibodies directed against the E2 envelope glycoprotein of hepatitis C virus. J Virol 83:12473–82. doi: 10.1128/JVI.01138-09 19759151 10.1128/JVI.01138-09 PMC2786766

[31] Tarr AW, Owsianka AM, Timms JM, McClure CP, Brown RJ, Hickling TP, et al (2006) Characterization of the hepatitis C virus E2 epitope defined by the broadly neutralizing monoclonal antibody AP33. Hepatology 43:592–601. doi: 10.1002/hep.21088 16496330 10.1002/hep.21088

[32] Owsianka A, Tarr AW, Juttla VS, Lavillette D, Bartosch B, Cosset FL, et al (2005) Monoclonal antibody AP33 defines a broadly neutralizing epitope on the hepatitis C virus E2 envelope glycoprotein. J Virol 79:11095–104. doi: 10.1128/JVI.79.17.11095-11104.2005 16103160 10.1128/JVI.79.17.11095-11104.2005 PMC1193588

[33] Law M, Maruyama T, Lewis J, Giang E, Tarr AW, Stamataki Z, et al (2008) Broadly neutralizing antibodies protect against hepatitis C virus quasispecies challenge. Nat Med 14:25–7. doi: 10.1038/nm1698 18064037 10.1038/nm1698

[34] Bailey JR, Wasilewski LN, Snider AE, El-Diwany R, Osburn WO, Keck Z, et al (2015) Naturally selected hepatitis C virus polymorphisms confer broad neutralizing antibody resistance. J Clin Invest 125:437–47. doi: 10.1172/JCI78794 25500884 10.1172/JCI78794 PMC4382262

[35] Giang E, Dorner M, Prentoe JC, Dreux M, Evans MJ, Bukh J, et al (2012) Human broadly neutralizing antibodies to the envelope glycoprotein complex of hepatitis C virus. Proc Natl Acad Sci U S A 109:6205–10. doi: 10.1073/pnas.1114927109 22492964 10.1073/pnas.1114927109 PMC3341081

[36] Potter JA, Owsianka AM, Jeffery N, Matthews DJ, Keck ZY, Lau P, et al (2012) Toward a hepatitis C virus vaccine: the structural basis of hepatitis C virus neutralization by AP33, a broadly neutralizing antibody. J Virol 86:12923–32. doi: 10.1128/JVI.02052-12 22993159 10.1128/JVI.02052-12 PMC3497650

[37] Drummer HE, Boo I, Maerz AL, Poumbourios P. (2006) A conserved Gly436-Trp-Leu-Ala-Gly-Leu-Phe-Tyr motif in hepatitis C virus glycoprotein E2 is a determinant of CD81 binding and viral entry. J Virol 80:7844–53. doi: 10.1128/JVI.00029-06 16873241 10.1128/JVI.00029-06 PMC1563787

[38] Yagnik AT, Lahm A, Meola A, Roccasecca RM, Ercole BB, Nicosia A, et al (2000) A model for the hepatitis C virus envelope glycoprotein E2. Proteins 40:355–66. 10861927 10.1002/1097-0134(20000815)40:3<355::aid-prot20>3.0.co;2-k

[39] Roccasecca R, Ansuini H, Vitelli A, Meola A, Scarselli E, Acali S, et al (2003) Binding of the hepatitis C virus E2 glycoprotein to CD81 is strain specific and is modulated by a complex interplay between hypervariable regions 1 and 2. J Virol 77:1856–67. doi: 10.1128/JVI.77.3.1856-1867.2003 12525620 10.1128/JVI.77.3.1856-1867.2003 PMC140892

[40] Op De Beeck A, Voisset C, Bartosch B, Ciczora Y, Cocquerel L, Keck Z, et al (2004) Characterization of functional hepatitis C virus envelope glycoproteins. J Virol 78:2994–3002. doi: 10.1128/JVI.78.6.2994-3002.2004 14990718 10.1128/JVI.78.6.2994-3002.2004 PMC353750

[41] Vieyres G, Thomas X, Descamps V, Duverlie G, Patel AH, Dubuisson J. (2010) Characterization of the envelope glycoproteins associated with infectious hepatitis C virus. J Virol 84:10159–68. doi: 10.1128/JVI.01180-10 20668082 10.1128/JVI.01180-10 PMC2937754

[42] Viral Hepatitis-Statistics and Surveillance 2015. Available from: http://www.cdc.gov/hepatitis/statistics/.

[43] Prentoe J, Velazquez-Moctezuma R, Foung SK, Law M, Bukh J. (2016) Hypervariable region 1 shielding of hepatitis C virus is a main contributor to genotypic differences in neutralization sensitivity. Hepatology 64:1881–92. doi: 10.1002/hep.28705 27351277 10.1002/hep.28705 PMC5115964

[44] Ralston R, Thudium K, Berger K, Kuo C, Gervase B, Hall J, et al (1993) Characterization of hepatitis C virus envelope glycoprotein complexes expressed by recombinant vaccinia viruses. J Virol 67:6753–61. 8411378 10.1128/jvi.67.11.6753-6761.1993 PMC238116

[45] Logan M, Law J, Wong JA, Hockman D, Landi A, Chen C, et al (2017) Native Folding of a Recombinant gpE1/gpE2 Heterodimer Vaccine Antigen from a Precursor Protein Fused with Fc IgG. J Virol 91. 10.1128/JVI.01552-16 PMC5165201 27795422

[46] Drummer HE, Boo I, Poumbourios P. (2007) Mutagenesis of a conserved fusion peptide-like motif and membrane-proximal heptad-repeat region of hepatitis C virus glycoprotein E1. J Gen Virol 88:1144–8. doi: 10.1099/vir.0.82567-0 17374757 10.1099/vir.0.82567-0

[47] Flint M, Thomas JM, Maidens CM, Shotton C, Levy S, Barclay WS, et al (1999) Functional analysis of cell surface-expressed hepatitis C virus E2 glycoprotein. J Virol 73:6782–90. 10400776 10.1128/jvi.73.8.6782-6790.1999 PMC112763

[48] Perez-Berna AJ, Moreno MR, Guillen J, Bernabeu A, Villalain J. (2006) The membrane-active regions of the hepatitis C virus E1 and E2 envelope glycoproteins. Biochemistry 45:3755–68. doi: 10.1021/bi0523963 16533059 10.1021/bi0523963

[49] Tong Y, Chi X, Yang W, Zhong J. (2017) Functional analysis of HCV envelope protein E1 using a trans-complementation system reveals a dual role of a putative fusion peptide of E1 in both HCV entry and morphogenesis. J Virol 91:e02468–16 doi: 10.1128/JVI.02468-16 28100619 10.1128/JVI.02468-16 PMC5355622

[50] Kielian M, Rey FA. (2006) Virus membrane-fusion proteins: more than one way to make a hairpin. Nat Rev Microbiol 4:67–76. doi: 10.1038/nrmicro1326 16357862 10.1038/nrmicro1326 PMC7097298

[51] Harrison SC. (2015) Viral membrane fusion. Virology 479–480:498–507. doi: 10.1016/j.virol.2015.03.043 25866377 10.1016/j.virol.2015.03.043 PMC4424100

[52] Li HF, Huang CH, Ai LS, Chuang CK, Chen SS. (2009) Mutagenesis of the fusion peptide-like domain of hepatitis C virus E1 glycoprotein: involvement in cell fusion and virus entry. J Biomed Sci 16:89 doi: 10.1186/1423-0127-16-89 19778418 10.1186/1423-0127-16-89 PMC2759930

[53] Owsianka A, Clayton RF, Loomis-Price LD, McKeating JA, Patel AH. (2001) Functional analysis of hepatitis C virus E2 glycoproteins and virus-like particles reveals structural dissimilarities between different forms of E2. J Gen Virol 82:1877–83. doi: 10.1099/0022-1317-82-8-1877 11457993 10.1099/0022-1317-82-8-1877

[54] Sabahi A, Uprichard SL, Wimley WC, Dash S, Garry RF. (2014) Unexpected structural features of the hepatitis C virus envelope protein 2 ectodomain. J Virol 88:10280–8. doi: 10.1128/JVI.00874-14 24991010 10.1128/JVI.00874-14 PMC4178838

[55] Keck ZY, Li TK, Xia J, Gal-Tanamy M, Olson O, Li SH, et al (2008) Definition of a conserved immunodominant domain on hepatitis C virus E2 glycoprotein by neutralizing human monoclonal antibodies. J Virol 82:6061–6. doi: 10.1128/JVI.02475-07 18400849 10.1128/JVI.02475-07 PMC2395155

[56] Pierce BG, Keck ZY, Lau P, Fauvelle C, Gowthaman R, Baumert TF, et al (2016) Global mapping of antibody recognition of the hepatitis C virus E2 glycoprotein: Implications for vaccine design. Proc Natl Acad Sci U S A 113:e6946–54. 10.1073/pnas.1614942113 PMC5111724 27791171

[57] Krey T, Meola A, Keck ZY, Damier-Piolle L, Foung SK, Rey FA. (2013) Structural basis of HCV neutralization by human monoclonal antibodies resistant to viral neutralization escape. PLoS Pathog 9:e1003364 doi: 10.1371/journal.ppat.1003364 23696737 10.1371/journal.ppat.1003364 PMC3656090

[58] Sautto G, Tarr AW, Mancini N, Clementi M. (2013) Structural and antigenic definition of hepatitis C virus E2 glycoprotein epitopes targeted by monoclonal antibodies. Clin Dev Immunol 2013:450963 doi: 10.1155/2013/450963 23935648 10.1155/2013/450963 PMC3722892

[59] Prentoe J, Jensen TB, Meuleman P, Serre SB, Scheel TK, Leroux-Roels G, et al (2011) Hypervariable region 1 differentially impacts viability of hepatitis C virus strains of genotypes 1 to 6 and impairs virus neutralization. J Virol 85:2224–34. doi: 10.1128/JVI.01594-10 21123377 10.1128/JVI.01594-10 PMC3067759

[60] Carlsen TH, Pedersen J, Prentoe JC, Giang E, Keck ZY, Mikkelsen LS, et al (2014) Breadth of neutralization and synergy of clinically relevant human monoclonal antibodies against HCV genotypes 1a, 1b, 2a, 2b, 2c, and 3a. Hepatology 60:1551–62. doi: 10.1002/hep.27298 25043937 10.1002/hep.27298 PMC4415877

[61] Urbanowicz RA, McClure CP, Brown RJ, Tsoleridis T, Persson MA, Krey T, et al (2015) A Diverse Panel of Hepatitis C Virus Glycoproteins for Use in Vaccine Research Reveals Extremes of Monoclonal Antibody Neutralization Resistance. J Virol 90:3288–301. doi: 10.1128/JVI.02700-15 26699643 10.1128/JVI.02700-15 PMC4794667

[62] McCaffrey K, Boo I, Poumbourios P, Drummer HE. (2007) Expression and characterization of a minimal hepatitis C virus glycoprotein E2 core domain that retains CD81 binding. J Virol 81:9584–90. doi: 10.1128/JVI.02782-06 17581991 10.1128/JVI.02782-06 PMC1951388

[63] McCaffrey K, Gouklani H, Boo I, Poumbourios P, Drummer HE. (2011) The variable regions of hepatitis C virus glycoprotein E2 have an essential structural role in glycoprotein assembly and virion infectivity. J Gen Virol 92:112–21. doi: 10.1099/vir.0.026385-0 20926639 10.1099/vir.0.026385-0

[64] Fraser J, Boo I, Poumbourios P, Drummer HE. (2011) Hepatitis C virus (HCV) envelope glycoproteins E1 and E2 contain reduced cysteine residues essential for virus entry. J Biol Chem 286:31984–92. doi: 10.1074/jbc.M111.269605 21768113 10.1074/jbc.M111.269605 PMC3173156

[65] McCaffrey K, Boo I, Tewierek K, Edmunds ML, Poumbourios P, Drummer HE. (2012) Role of conserved cysteine residues in hepatitis C virus glycoprotein e2 folding and function. J Virol 86:3961–74. doi: 10.1128/JVI.05396-11 22278231 10.1128/JVI.05396-11 PMC3302498

[66] Albecka A, Montserret R, Krey T, Tarr AW, Diesis E, Ball JK, et al (2011) Identification of new functional regions in hepatitis C virus envelope glycoprotein E2. J Virol 85:1777–92. doi: 10.1128/JVI.02170-10 21147916 10.1128/JVI.02170-10 PMC3028898

[67] Yanagi M, Purcell RH, Emerson SU, Bukh J. (1997) Transcripts from a single full-length cDNA clone of hepatitis C virus are infectious when directly transfected into the liver of a chimpanzee. Proc Natl Acad Sci U S A 94:8738–43. 9238047 10.1073/pnas.94.16.8738 PMC23104

[68] Paes C, Ingalls J, Kampani K, Sulli C, Kakkar E, Murray M, et al (2009) Atomic-level mapping of antibody epitopes on a GPCR. J Am Chem Soc 131:6952–4. doi: 10.1021/ja900186n 19453194 10.1021/ja900186n PMC2943208

[69] Selvarajah S, Sexton NR, Kahle KM, Fong RH, Mattia KA, Gardner J, et al (2013) A neutralizing monoclonal antibody targeting the acid-sensitive region in chikungunya virus E2 protects from disease. PLoS Negl Trop Dis 7:e2423 doi: 10.1371/journal.pntd.0002423 24069479 10.1371/journal.pntd.0002423 PMC3772074

[70] Drummer HE, Wilson KA, Poumbourios P. (2005) Determinants of CD81 dimerization and interaction with hepatitis C virus glycoprotein E2. Biochem Biophys Res Commun 328:251–7. doi: 10.1016/j.bbrc.2004.12.160 15670777 10.1016/j.bbrc.2004.12.160

[71] Ruwona TB, Giang E, Nieusma T, Law M. (2014) Fine mapping of murine antibody responses to immunization with a novel soluble form of hepatitis C virus envelope glycoprotein complex. J Virol 88:10459–71. doi: 10.1128/JVI.01584-14 24965471 10.1128/JVI.01584-14 PMC4178869

[72] Salvador B, Zhou Y, Michault A, Muench MO, Simmons G. (2009) Characterization of Chikungunya pseudotyped viruses: Identification of refractory cell lines and demonstration of cellular tropism differences mediated by mutations in E1 glycoprotein. Virology 393:33–41. doi: 10.1016/j.virol.2009.07.013 19692105 10.1016/j.virol.2009.07.013 PMC2760448

[73] Simmons G, Reeves JD, Rennekamp AJ, Amberg SM, Piefer AJ, Bates P. (2004) Characterization of severe acute respiratory syndrome-associated coronavirus (SARS-CoV) spike glycoprotein-mediated viral entry. Proc Natl Acad Sci U S A 101:4240–5. doi: 10.1073/pnas.0306446101 15010527 10.1073/pnas.0306446101 PMC384725

[74] Naldini L, Blomer U, Gallay P, Ory D, Mulligan R, Gage FH, et al (1996) In vivo gene delivery and stable transduction of nondividing cells by a lentiviral vector. Science 272:263–7. 8602510 10.1126/science.272.5259.263

[75] Connor RI, Chen BK, Choe S, Landau NR. (1995) Vpr is required for efficient replication of human immunodeficiency virus type-1 in mononuclear phagocytes. Virology 206:935–44. doi: 10.1006/viro.1995.1016 7531918 10.1006/viro.1995.1016

[76] Bartosch B, Dubuisson J, Cosset FL. (2003) Infectious hepatitis C virus pseudo-particles containing functional E1-E2 envelope protein complexes. J Exp Med 197:633–42. doi: 10.1084/jem.20021756 12615904 10.1084/jem.20021756 PMC2193821

[77] Pickett BE, Sadat EL, Zhang Y, Noronha JM, Squires RB, Hunt V, et al (2012) ViPR: an open bioinformatics database and analysis resource for virology research. Nucleic Acids Res 40:D593–8. doi: 10.1093/nar/gkr859 22006842 10.1093/nar/gkr859 PMC3245011

[78] Laskowski RA, Swindells MB. (2011) LigPlot+: multiple ligand-protein interaction diagrams for drug discovery. J Chem Inf Model 51:2778–86. doi: 10.1021/ci200227u 21919503 10.1021/ci200227u

