# Supplementary figures and images for: Probing the antigenicity of hepatitis C virus envelope glycoprotein complex by high-throughput mutagenesis
Source: PLoS Pathog. 2017 Dec 18;13(12):e1006735. doi: 10.1371/journal.ppat.1006735 (PMC5749897; doi:10.1371/journal.ppat.1006735)

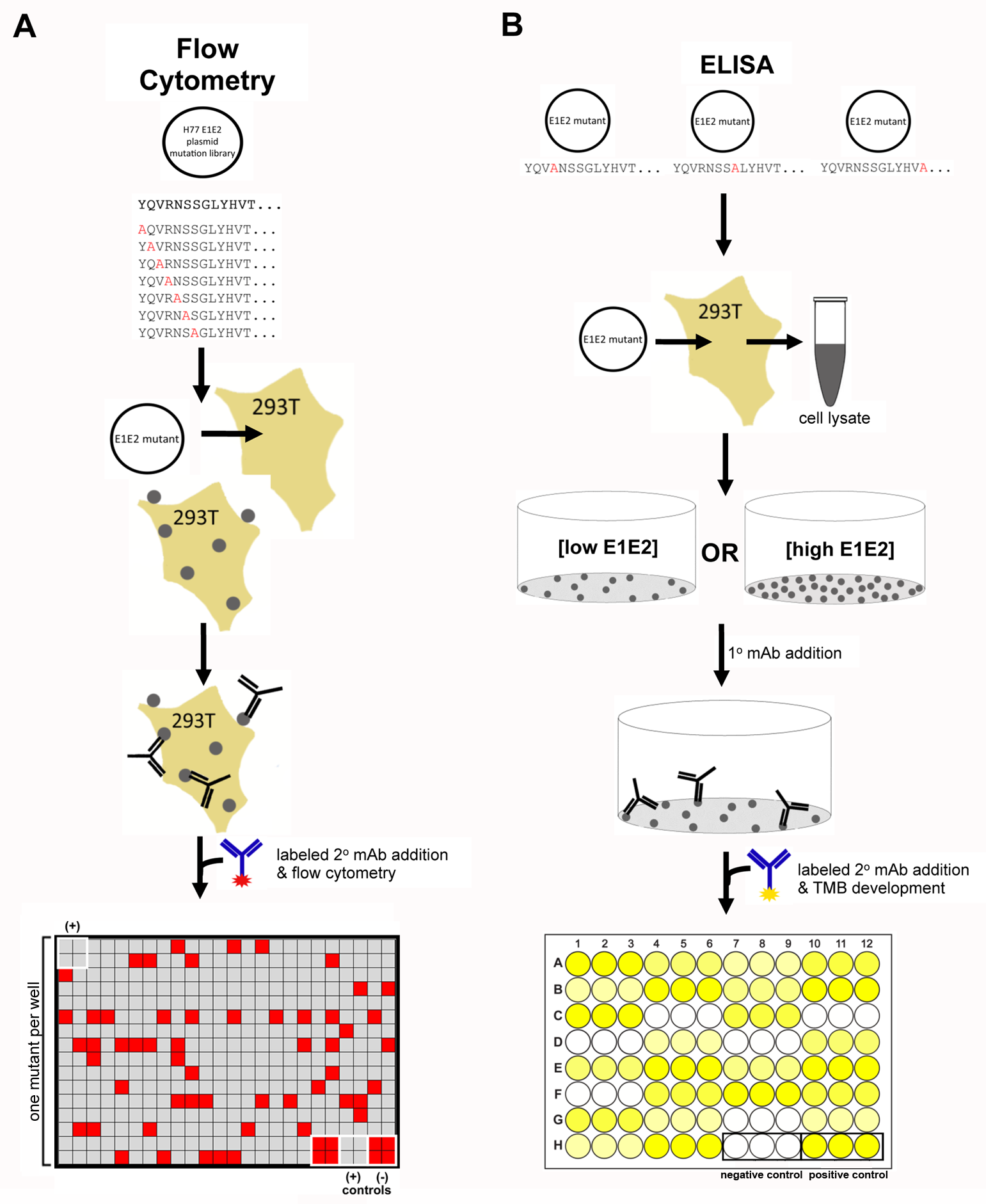

Supplement: S1 Fig — Shotgun mutagenesis followed by whole cell analysis using flow cytometry offers an alternative to ELISA. (A) High-throughput shotgun mutagenesis was used to create a plasmid mutant library for the entirety of E1E2, with each clone having a defined alanine mutation. Prior to screening the mutant library, each primary mAb was tested against wild-type E1E2 to determine optimal mAb concentration and conditions to achieve high signal to background ratio. HEK-293T cells were transfected with individual mutants from the library and subsequently fixed and permeabilized. Each mAb was tested against each mutant in the library by flow cytometry using a fluorescent secondary mAb to detect immunoreactivity. Background signal from mock-transfected cells was subtracted and the signals were normalized to the wild-type E1E2 positive control [26]. (B) Site-directed mutagenesis was performed on individual conserved residues of E1E2. Mutant E1E2-containing plasmids were transfected into HEK-293T cells and the cell lysate was added to lectin-coated ELISA plates at either high (1:5) or low (1:50) concentration. The panel of mAbs was tested against the mutant cell lysate using an HRP-conjugated secondary antibody and TMB substrate to detect reactivity. Background signal from mock-transfected cell lysate was subtracted and the signal was normalized to the wild-type E1E2 positive control. (TIF) [file ppat.1006735.s001.tif]

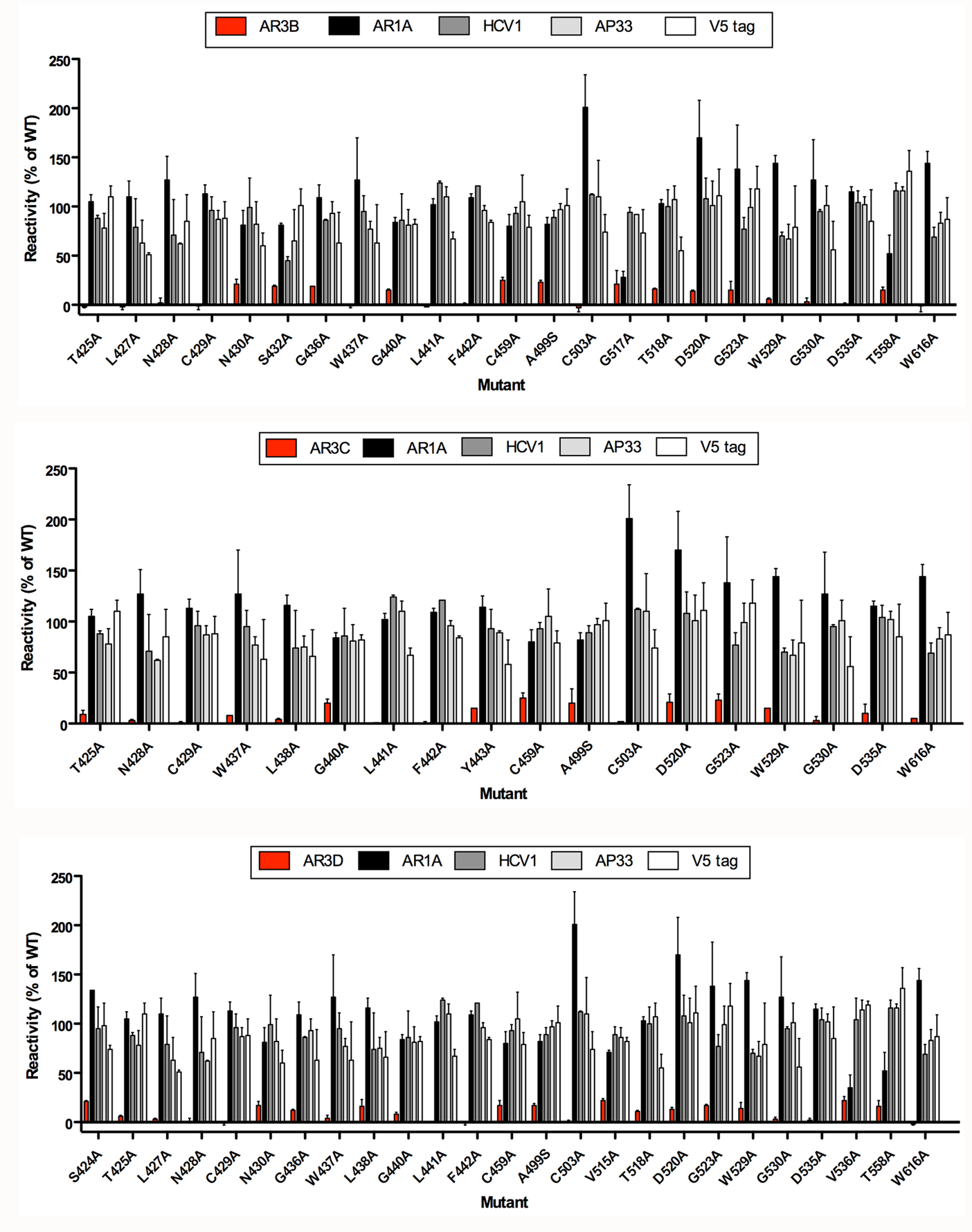

Supplement: S2 Fig — Data shown are the mutations resulting in ≤25% mean reactivity, expressed as percent of wild-type AR3B-D mAbs, but >75% of at least one control mAb, using the E1E2 mutant library and flow cytometry analysis (see Fig 6A for AR3A data). Mutants resulting in poor E1E2 expression (<40%) as determined by the C-terminal V5 tag and those implicated in global misfolding were removed. Binding assays were performed twice with the range indicated. (TIF) [file ppat.1006735.s002.tif]

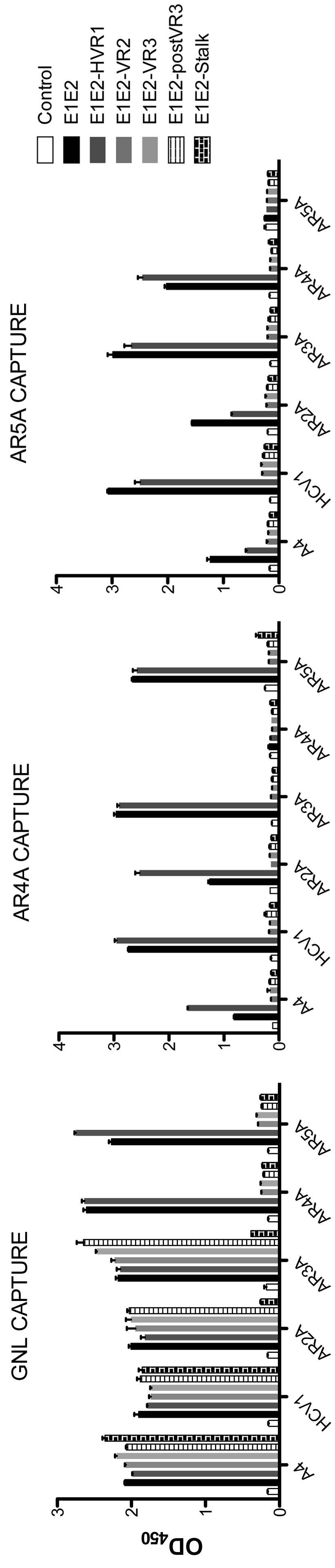

Supplement: S3 Fig — The importance of variable regions (HVR1, R2, VR3, post-VR3) and stalk of E2 for E1E2 complex formation was tested by ELISA. E1E2 from HEK-293T cell lysates (undiluted) co-transfected with deletion mutants lacking these regions and pAdv plasmid was captured using GNL, AR4A or AR5A (as indicated) and evaluated for binding using a panel of antibodies. Full-length E1E2 without deletions (E1E2) and pAdv plasmid (Control) were used as positive and negative control, respectively. (TIF) [file ppat.1006735.s003.tif]

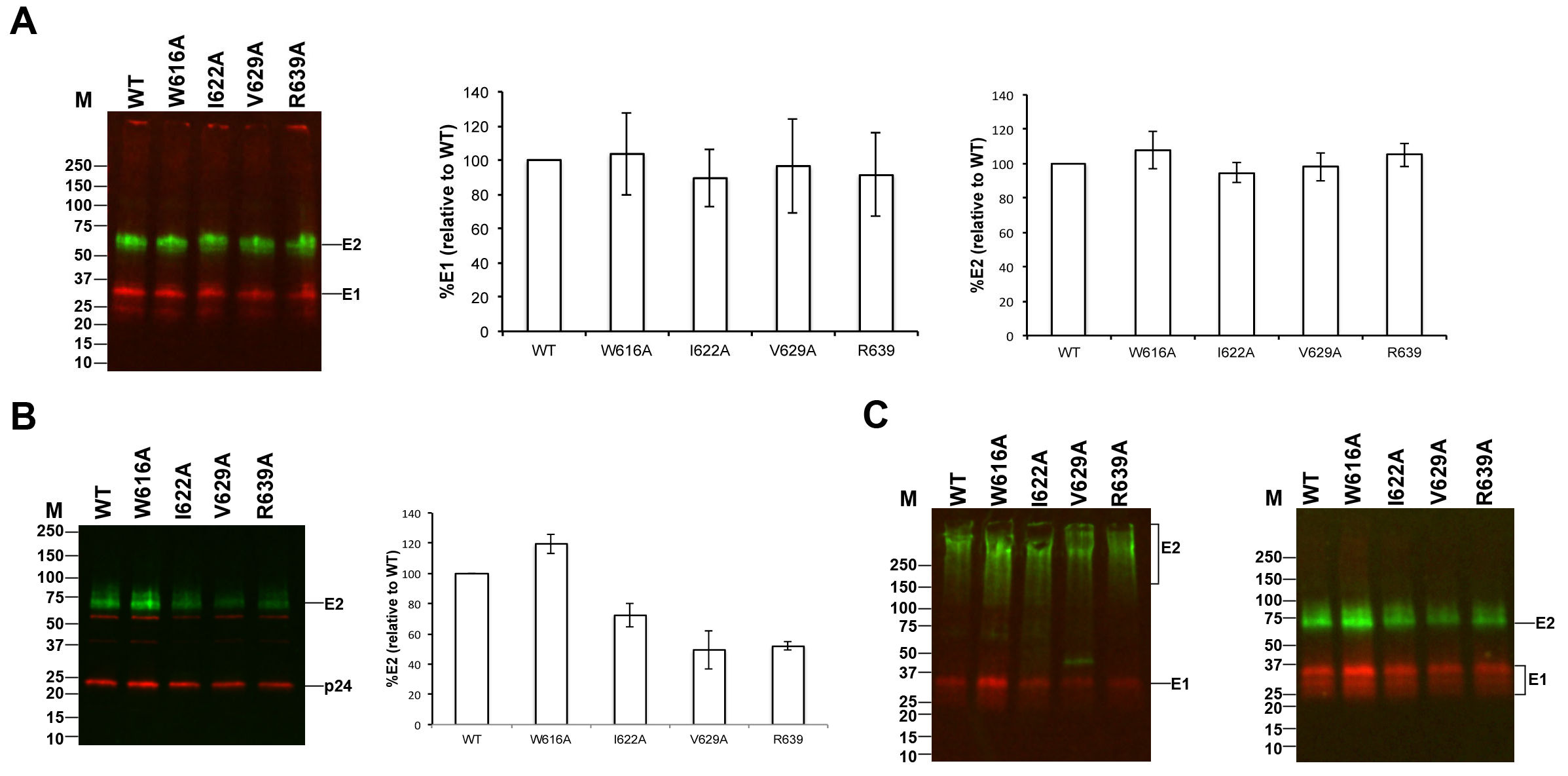

Supplement: S4 Fig — HCVpp was generated by transfecting 293T cells with plasmids expressing wild-type or back layer E1E2 mutant (W616A, I622A, V629A and R639A). A) Transfected cell lysates were analyzed by reducing SDS-PAGE and immunoblotting using biotinylated mAb A4 for E1 (red) and mAb HCV1 for E2 (green). E1 and E2 in mutants were quantified as a percentage of wild-type levels (middle and right panels). B) Purified virions were analyzed by reducing SDS-PAGE and immunoblotting using mAb HCV1 for E2 (green) and anti-p24 for HIV-1-p24 (red). Percentage of E2 (relative to wild-type) was normalized to corresponding p24 levels (right panel). C) Purified virions were analyzed by non-reducing (left panel) and reducing (right panel) SDS-PAGE and immunoblotting using biotinylated mAb A4 for E1 (red) and mAb HCV1 for E2 (green). M, Molecular Marker; WT, wild-type. Experiments were performed in duplicate. (TIF) [file ppat.1006735.s004.tif]
